# Explainable and interpretable models for predicting early-onset hypertension in the Tlalpan 2020 cohort

**DOI:** 10.3389/fdgth.2026.1805107

**Published:** 2026-06-02

**Authors:** Guadalupe Gutiérrez-Esparza, Mireya Martínez-García, Luis M. Amezcua-Guerra, Martín Montes Rivera, Enrique Hernández-Lemus

**Affiliations:** 1 “Researcher for Mexico” Program, Secretaría de Ciencia, Humanidades, Tecnología e Innovación (SECIHTI), Mexico City, Mexico; 2 Diagnostic and Treatment Services, Instituto Nacional de Cardiología Ignacio Chávez, Mexico City, Mexico; 3 Department of Immunology, Instituto Nacional de Cardiología Ignacio Chávez, Mexico City, Mexico; 4 Dental Public Health Department, Division of Graduate Studies and Research, School of Dentistry, Universidad Nacional Autónoma de México, Mexico City, Mexico; 5 Research and postgraduate studies department, Universidad Politécnica de Aguascalientes, Aguascalientes, Ags., Mexico; 6 Computational Genomics Division, Instituto Nacional de Medicina Genómica, Mexico City, Mexico

**Keywords:** anxiety, energy drink consumption, family history, foods consumption, high-fat, hypertension, particle swarm optimization programming, sedentary lifestyle

## Abstract

**Background:**

Early-onset hypertension results from complex interactions among demographic, lifestyle, metabolic, and psychosocial factors. While machine learning models can predict hypertension with relative accuracy, their lack of interpretability limits their clinical utility.

**Methods:**

Using a nested case-control design based on the Tlalpan 2020 prospective cohort, a 10-year study of clinically healthy adults in Mexico City, this study applies DSRegPSOP, a symbolic regression approach, to develop interpretable mathematical models. The dataset included demographic, clinical, biochemical, lifestyle, and sleep-related variables. We addressed class imbalance using oversampling and SMOTE-based strategies, and evaluated model performance with accuracy, sensitivity, specificity, F1-score, and AUC-ROC.

**Results:**

DSRegPSOP produced compact analytical expressions with predictive performance comparable to state-of-the-art machine learning algorithms while preserving interpretability. The resulting models reveal clinically meaningful predictors of early-onset hypertension.

**Conclusion:**

DSRegPSOP provides a transparent and interpretable model for hypertension risk assessment that shows promising potential to support early prevention strategies, pending external validation on independent cohorts.

## 1 Introduction

Hypertension is a multifactorial condition whose early development results from the interaction of biological, behavioral, and environmental determinants. Understanding these determinants requires high-quality longitudinal data capable of capturing lifestyle behaviors, clinical biomarkers, anthropometric trajectories, psychosocial factors, and metabolic alterations long before the clinical onset of the disease. In Mexico, the Tlalpan 2020 cohort was specifically designed to address this gap, providing one of the most comprehensive prospective datasets for studying incident hypertension in clinically healthy adults.

The Tlalpan 2020 cohort is a 10-year, population-based, longitudinal study conducted in Mexico City, which recruited clinically healthy adults aged 20–50 years and follows them every 2 years to determine factors associated with the incidence of systemic hypertension. The cohort includes standardized measurements of blood pressure, anthropometry, biochemical markers, lifestyle exposures, sleep quality, anxiety, and 24-h urinary sodium and potassium excretion. These measurements were collected following clinical protocols established at the Instituto Nacional de Cardiología Ignacio Chávez, creating a high-quality epidemiological resource for predictive modeling.

Developing predictive models for early-onset hypertension requires integrating diverse demographic, lifestyle, and clinical variables. Although several machine learning techniques—such as decision trees, random forests, support vector machines, and neural networks—have been widely used for cardiovascular risk prediction, many of these methods lack interpretability (1). This is a critical limitation in medical contexts, where understanding the contribution of each predictor is essential for clinical decision-making. Therefore, interpretable modeling approaches capable of providing explicit mathematical relationships between risk factors and outcomes are needed.

On the other hand, symbolic regression is an approach to determining the mathematical expression that best fits a dataset to predict a given outcome (2). Symbolic regression offers several advantages over traditional machine learning techniques, including interpretability, flexibility, and the ability to discover novel relationships between variables (3).

Furthermore, symbolic regression has been successfully applied in medical applications, such as the prediction of heart failure deaths (4), to predict primary aldosteronism and apparent treatment-resistant in hypertension (5), in the generation of medical scoring models with continual learning (6), in the detection of hepatitis C and the disease progression (7), in morality prediction in surgery (8), assessing perioperative risks in elderly surgical population (9), among others. There are several techniques for symbolic regression, but all of them are regression-based, expression-tree-based, physics-inspired, or mathematics-inspired (2). Genetic programming (GP) is the most common technique to perform symbolic regression (10). GP is an evolutionary algorithm that mimics natural selection to evolve computer programs that solve a problem (11).

In GP, a population of candidate solutions is evolved over generations using evolutionary algorithm operators such as selection, crossover, and mutation. The fitness of each program is evaluated based on its ability to solve the problem. Over generations, the population evolves towards better solutions. Nevertheless, GP is limited by its complex representations and evolutionary mechanisms, which demand high computational resources for high-order computations, especially for large populations (12). Alternatively, swarm intelligence (SI) algorithms involve lower-order computations compared with evolutionary algorithms and have been successfully applied to numerical optimization problems (13). However, their application in large-scale, complex search spaces, such as those explored in symbolic regression, is inefficient, as they are likely to converge to local optima (14).

In this work, we propose using a novel swarm intelligence algorithm, DSRegPSOP (Dynamic Sphere Regrouping Particle Swarm Optimization Programming), which implements DSRegPSO for symbolic regression. DSRegPSO is based on the Particle Swarm Optimization (PSO) algorithm proposed initially in Kennedy and Eberhart (15), which is inspired by the social behavior of birds and fish. In PSO, a population of particles (candidate solutions) moves through the search space, adjusting their positions based on their own experience and the experience of their neighbors. The goal is to find the optimal solution by iteratively updating the positions and velocities of the particles.

The stagnation problem in local optimal regions is addressed in DSRegPSO introducing two mechanisms: a regrouping system that reinvigorates the swarm by continually resetting position of particles that are under a threshold distance from the global best particle controlled with an sphere that adapts its diameter allowing exploration and exploitation of the search space, and a momentum effect that make particles return to explore the search space if they travel to the limits of it. Moreover, DSRegPSO adapts speed and inertial components based on sphere diameter, maximizing exploration and exploitation when it is more needed (16). Furthermore, the use of DSRegPSOP in symbolic regression produces similar results in classification and regression problems to those obtained with GP, or with different models like Random Forest, Tree Regression, Partial Least Square Regression, Ridge Regression, XGBoost, Neuro-Fuzzy Systems, and Artificial Neural Networks (ANNs), among others, in publicly available datasets (14).

Here we used DSRegPSOP to develop mathematical models for predicting early-onset hypertension in the Tlalpan 2020 cohort. With this approach, we aim to create interpretable symbolic regression models with accuracy, sensitivity, and specificity comparable to those of standard machine learning algorithms and artificial neural networks, while providing explainable models that are easier to implement and diagnose, offering insights into the factors contributing to hypertension development, ultimately aiding early diagnosis and intervention strategies.

**Figure 1:**
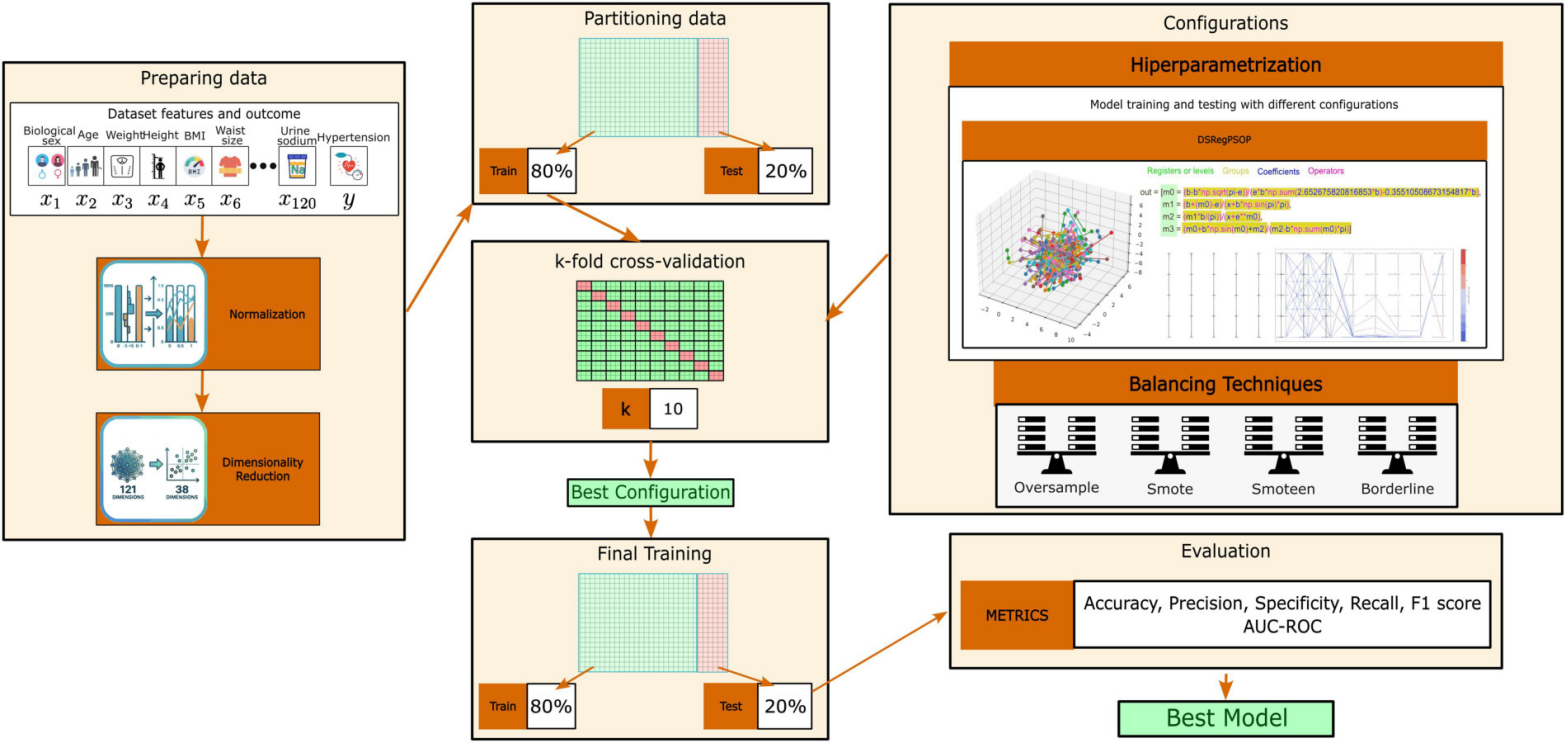
DSRegPSOP for predicting early-onset hypertension in the Tlalpan 2020 cohort.

The methodology follows a six-stage pipeline: (1) Z-score normalization of the entire dataset; (2) Pearson correlation-based dimensionality reduction for feasible computations, retaining features with coefficient ≥0.05, reducing the feature space from 121 to 38 dimensions; (3) partitioning into training (80%) and held-out test (20%) sets; (4) 10-fold cross-validation grid search on the training set to optimize DSRegPSOP hyperparameters and balancing strategy; (5) final model training on the complete training set, during which DSRegPSOP implicitly selected between 5 and 25 features via its evolutionary symbolic regression process; and (6) final evaluation on the held-out test set, providing an unbiased assessment of predictive performance. Figure 1 shows an overview of the proposed methodology using DSRegPSOP for predicting early-onset hypertension in the Tlalpan 2020 cohort.

## 2 Materials and methods

### 2.1 Data

The data used in this study come from the Tlalpan 2020 cohort (17), a prospective, population-based study designed to identify determinants of early-onset systemic hypertension in clinically healthy adults in Mexico City. Participants were evaluated at the Instituto Nacional de Cardiología Ignacio Chávez following standardized protocols for anthropometry, biochemical testing, and blood pressure assessment. All participants provided written informed consent before enrollment. The study protocol and procedures were approved by the Research Ethics Committee of the National Institute of Cardiology Ignacio Chávez (INCICH) under code 13-802. For the present analysis, a subset of 750 individuals with complete information was included.

Among these individuals, 150 developed hypertension during follow-up, while 600 remained normotensive and were matched by age and sex, forming the case–control dataset used for model development. This corresponds to a class distribution of 150 hypertensive and 600 normotensive individuals (1:4 ratio).

This case–control sampling strategy was designed for model development; therefore, the proportion of hypertensive individuals in this subset does not represent the true incidence of hypertension in the original cohort.

The dataset includes 121 normalized variables representing multiple domains relevant to hypertension research. Prior to model development, continuous features were standardized using z-score normalization (mean = 0, standard deviation = 1) during the dataset preprocessing stage, while binary variables were retained in their original scale.

Demographic variables include age and sex. Anthropometric measurements—including weight, height, Body Mass Index (BMI), waist circumference, and basal systolic and basal diastolic blood pressure (SBP and DBP, as measured at the initial time, prior to the development or non-development of hypertension)—were obtained following standardized clinical protocols (18). Biochemical variables such as fasting glucose, lipid profile, triglycerides, serum sodium, and creatinine were measured using validated enzymatic and ion-selective electrode methods. Hematological parameters—including hemoglobin, hematocrit, platelet count, red blood cell indices, and leukocyte differentials—were obtained through automated analysis on a Beckman Coulter LH-series hematology platform, operated under standardized calibration and quality-control procedures. All laboratory determinations were performed in a facility accredited by the Mexican Accreditation Entity (EMA, A.C.) under the NMX-EC-15189-IMNC-2015 / ISO 15189:2012 standard, ensuring technical competence, traceability, and adherence to internationally recognized requirements for analytical reliability. Urinary biomarkers were assessed from 24-h urine collections when available.

In addition to clinical and biochemical measurements, the dataset contains information related to lifestyle, sleep, psychological factors and physical activity. Participants reported their smoking and alcohol habits, weekly activity patterns and self-perceived stress levels. Sleep-related variables were obtained through the validated Spanish adaptation of the Medical Outcomes Study Sleep Scale (MOS-Sleep), which evaluates multiple aspects of nocturnal rest, including sleep onset, duration, efficiency and daytime somnolence. Each sleep dimension yields continuous scores on a 0–100 scale, with higher values reflecting a greater presence of the characteristic assessed. A global indicator summarizing overall sleep disturbances was also computed in accordance with the scoring guidelines of the instrument (19–22).

Psychological stress and anxiety were assessed using the Spanish validated version of the State–Trait Anxiety Inventory (STAI) (23). This questionnaire consists of two independent components that distinguish between situational, transient anxiety responses and more stable anxiety patterns. Both sections produce numerical scores that can be used continuously or categorized to reflect different levels of anxiety severity.

Physical activity was characterized using the International Physical Activity Questionnaire (IPAQ) (24), which estimates weekly energy expenditure in metabolic equivalents. Based on the standard IPAQ scoring system, participants were classified into low, moderate or high activity groups. These categories were also transformed into binary indicators to facilitate their incorporation into the machine-learning and symbolic-regression analyses.

The outcome variable, Hypertension, was defined as a binary indicator (0 = normotensive, 1 = hypertensive). Hypertension status was determined according to standard clinical criteria (systolic blood pressure ≥140 mmHg, diastolic blood pressure ≥90 mmHg, or use of antihypertensive medication) during follow-up of participants initially recruited as normotensive adults aged 20–50 years. Basal systolic and basal diastolic blood pressure (SBP and DBP, as measured at the initial time, prior to the development or non-development of hypertension) measurements were recorded during the clinical assessment.

To further characterize the predictive structure of the dataset, three complementary feature selection methods were applied to compare the features selected by DSRegPSOP in their final models. LASSO (Least Absolute Shrinkage and Selection Operator), implemented as an L1-penalized logistic regression with a maximum of 1,000 iterations, was used to identify features with linear associations with the hypertension outcome, using the absolute value of the learned coefficients as importance scores. Complementarily, Mutual Information (MI) was estimated for each feature using the nearest-neighbor approach, which quantifies statistical dependency between each predictor and the outcome regardless of the functional form of their relationship, making it sensitive to non-linear associations that linear methods may overlook. Additionally, a Random Forest classifier with 100 estimators was trained on the dataset, and the Gini-based feature importances were extracted to capture non-linear interactions and variable contributions based on tree ensemble structure. All three methods were applied to the full dataset after z-score normalization, and the top-20 ranked features from each method were compared against the variables implicitly selected by DSRegPSOP across the five balancing strategies.

### 2.2 Dynamical sphere regrouping particle swarm optimization programming

The DSRegPSOP proposed in Montes Rivera et al. (14) is an adaptation of the DSRegPSO algorithm for symbolic regression tasks. In DSRegPSOP, each particle position represents a mathematical model encoded as subsequent $m_{j}$ mathematical equations disposed in $n_{l}$ levels, each equation is composed of $n_{g}$ groups of expressions separated with parentheses, each expression inside the parentheses contains $n_{v}$ coefficients or variables, and $n_{o}$ operators. The first-level equation depends on the dataset’s features, while subsequent levels may depend on the features or the output of a previous level. The symbolic expressions are obtained by transforming the position with d dimensions of n particles into mathematical expressions with $TS\;:\;X\in R^{n\times d}\to M\in S^{n\times ds}$ that associates position values a string space S with variable length ds depending on the obtained expression. The dimensions required for the position of each particle are calculated with Equation 1.

$$d=n_{l}\cdot [n_{g}\cdot (n_{o}+n_{v})+(n_{g}-1)]$$ (1)

The position of the particles is transformed into two integer matrices $M_{o}\in Z^{n\times d}$ and $M_{v}\in Z^{n\times d}$, for indexing the operators in the first one and variables or coefficients in the second one. Equations 2 and 3 show how to obtain them per i row for each particle.

$${\vec{M}}_{o,i}=\frac{{\vec{X}}_{i}-L_{l}}{L_{u}-L_{l}}\cdot (n_{o}-1)$$ (2)

$${\vec{M}}_{v,i}=\frac{{\vec{X}}_{i}-L_{l}}{L_{u}-L_{l}}\cdot (n_{v}-1+n_{c})$$ (3)

Where $L_{l}$ and $L_{u}$ are the lower and upper limits of the particle position, respectively, and $n_{c}$ is the number of positions in the list of variables assigned to numerical coefficients.

Once the matrices $M_{o}$ and $M_{v}$ are obtained, $m_{j}$ adds $L_{v}\left({\vec{M}}_{v,i}\right)$ or $L_{o}\left({\vec{M}}_{o,i}\right)$, depending on if the equation index is pair or odd. Where $L_{v}$ and $L_{o}$ are the lists of variables/coefficients and operators, respectively. Furthermore, when the index of the variable/coefficients exceeds $n_{v}$, it indicates that a numerical coefficient should be added to the expression. In this case, the coefficient is calculated using a linear mapping between the lower and upper limits of the particle position, scaled to the search space in ranges $\left[L_{l},L_{u}\right]$. The reamaping boundaries for the numerical coefficients are in ranges $\left[L_{lc},L_{uc}\right]$, determined with Equations 4 and 5.

$$L_{lc}=L_{l}+n_{v}\frac{L_{u}-L_{l}}{n_{v}+n_{c}}$$ (4)

$$L_{uc}=L_{u}$$ (5)

Then , the coefficient c is calculated with Equation 6.

$$c=(L_{u}-L_{l})\frac{{\vec{X}}_{i,l}-L_{lc}}{L_{uc}-L_{lc}}-L_{ui}$$ (6)

Finally, the transformation algorithm TS is described in Algorithm 1.

Algorithm 1 **Transformation.**

**Data:** $X,n_{l},n_{g},n_{o},n_{s},L_{l},L_{u},L_{v},L_{o}$

**Result:** M

1: $d=n_{l}\cdot [n_{g}\cdot (n_{o}+n_{v})+(n_{g}-1)]$;

2: M=[];

3: **for** i=1 to n **do**

4:   ${\vec{M}}_{i}=[]$;

5:   **for** j=1 to $n_{l}$ **do**

6:     $m_{j}=[]$;

7:     ${\vec{M}}_{o,i}=\frac{{\vec{X}}_{i}-L_{l}}{L_{u}-L_{l}}\cdot (n_{o}-1)$;

8:     ${\vec{M}}_{v,i}=\frac{{\vec{X}}_{i}-L_{l}}{L_{u}-L_{l}}\cdot (n_{v}-1+n_{c})$;

9:     **for** k=1 to $n_{g}$ **do**

10:       **if** k=0 **then**

11:        $m_{j}|\mathrm{add}{(}^{\prime }{(}^{\prime })$;

12:       **else** **if** $m_{j}[k-1]\neq^{\prime }{(}^{\prime }$ **then**

13:         $m_{j}\leftarrow \mathrm{add}{(}^{\prime }{(}^{\prime });$

14:       **end** **if**

15:       **for** $l=k\cdot \frac{\frac{d}{n_{l}}}{n_{g}}+j\cdot \frac{d}{n_{l}}$ to $(k+1)\cdot \frac{\frac{d}{n_{l}}}{n_{g}}+j\cdot \frac{d}{n_{l}}$ **do**

16:       **if** (l+1+mod(k,2)+mod(j,2))=0 **then**

17:          $m_{j}=\mathrm{add}(L_{o}({\vec{M}}_{o,i=l}))$;

18:         **else**

19:          **if** ${\vec{M}}_{v=i,l}<n_{v}$ **then**

20:           $m_{j}=\mathrm{add}(L_{v}({\vec{M}}_{v,i=l}))$;

21:          **else**

22:           $L_{lc}=L_{l}+n_{v}\frac{L_{u}-L_{l}}{n_{v}+n_{c}}$;

23:           $L_{uc}=L_{u}$;

24:           $c=(L_{u}-L_{l})\frac{{\vec{X}}_{i,l}-L_{lc}}{L_{uc}-L_{lc}}-L_{ui}$;

25:           $m_{j}\leftarrow \mathrm{add}(c)$;

26:          **end** **if**

27:         **end** **if**

28:        **end** **for**

29:       $m_{j}|\mathrm{add}{(}^{\prime }{)}^{\prime })$

30:      **end** **for**

31:     $M_{i}\leftarrow \mathrm{add}(m_{j})$;

32:   **end** **for**

33:  $M\leftarrow \mathrm{add}({\vec{M}}_{i})$;

34: **end for**

The optimization process of DSRegPSOP uses the algorithm of DSRegPSO detailed in Montes Rivera et al. (16). This variant of PSO introduces a dynamic sphere regrouping mechanism, controlled by the current sphere diameter δ, that regulates the inertial behavior ω and switches between exploration and exploitation as needed, making PSO suitable for large-scale optimization, such as in symbolic regression tasks.

DSRegPSO starts by determining ω, to equally distribute the maximum inertial momentum across $\frac{\delta }{\delta_{max}}$ and $\frac{S_{s}}{S_{\max}}$. Where $S_{s}$ is the current sphere expansion speed, and $S_{\max}$ is the maximum sphere expansion speed, $\delta_{max}$ is the maximum sphere diameter.

The speed equation of DSRegPSO has three components: the inertial component $\left(\frac{\delta }{\delta_{max}}+\frac{S_{s}}{S_{\max}}\right)$, the social component $\left(c_{1}\cdot {\vec{R}}_{1}\cdot \left({\vec{P}}_{G}-{\vec{X}}_{i}\right)\right)$, and the cognitive component $\left(c_{2}\cdot {\vec{R}}_{2}\cdot \left({\vec{P}}_{i}-{\vec{X}}_{i}\right)\right)$. Where ${\vec{X}}_{i}$ is the position of particle i, $c_{1}$ and $c_{2}$ are the social and cognitive coefficients, respectively, ${\vec{R}}_{1}$ and ${\vec{R}}_{2}$ are random vectors with values in the range [0,1]. ${\vec{P}}_{G}$ is the global best position and ${\vec{P}}_{i}$ is the personal best position. Therefore, the speed is updated at each iteration using the Equation 7.

$${\vec{V}}_{i}=\left[\frac{\delta }{\delta_{\max}}+\frac{S_{s}}{S_{\max}}\right]\cdot \omega \cdot {\vec{V}}_{i}+c_{1}\cdot {\vec{R}}_{1}\cdot ({\vec{P}}_{G}-{\vec{X}}_{i})+c_{2}\cdot {\vec{R}}_{2}\cdot ({\vec{P}}_{i}-{\vec{X}}_{i})$$ (7)

The speed limits in DSRegPSO are dynamically adjusted based on δ and $S_{s}$. The upper speed limit $LS_{u}$ is calculated as $LS_{u}=\left(\frac{\delta }{\delta_{max}}+\frac{S_{s}}{S_{\max}}\right)\cdot \omega$, while the lower speed limit $LS_{l}$ is set to the negative of the upper limit, i.e., $LS_{l}=-LS_{u}$. This dynamic adjustment allows particles to adapt their movement according to the exploration and exploitation needs of the search space.

Algorithm 2 shows the complete DSRegPSO; a more detailed explanation is included in Montes Rivera et al. (16).

Algorithm 2 **DSRegPSO.**

**Data:** $d,n,f({\vec{X}}_{i}),L_{l},L_{u},c_{1},c_{2},M_{\max},\lambda ,fd_{\max},fd_{\min},S_{\max},S_{\min},\zeta$

**Result:** ${\vec{P}}_{G}$

1: $LS_{ui}=\lambda (L_{l}-L_{u})$;

2: $LS_{u}=LS_{ui}$;

3: $LS_{l}=-LS_{u}$;

4: $\omega =\frac{M_{\max}}{2}$;

5: ${\vec{X}}_{i}\in [L_{l},L_{u}]$;

6: ${\vec{V}}_{i}=0$;

7: $C_{i}=f({\vec{X}}_{i})$;

8: ${\vec{G}}_{P,i}=C_{i}$;

9: ${\vec{P}}_{i}={\vec{X}}_{i}$;

10: $G_{B}=\min(G_{P,i})$;

11: ${\vec{P}}_{G}=\mathrm{argmin}f({\vec{X}}_{i})$;

12: $S_{s}=S_{\min}$;

13: $\delta_{\max}=\max({\vec{P}}_{G})\cdot fd_{\max}$;

14: $\delta_{\min}=\min({\vec{P}}_{G})\cdot fd_{\min}$;

15: $\delta =\delta_{\min}$;

16: it=0;

17: **while** $k<k_{\max}$ **or** $G_{B}\leq C_{d}$ **do**

18: ${\vec{V}}_{i}=\left[\frac{\delta }{\delta_{\max}}+\frac{S_{s}}{S_{\max}}\right]\cdot \omega \cdot {\vec{V}}_{i}+c_{1}\cdot {\vec{R}}_{1}\cdot ({\vec{P}}_{G}-{\vec{X}}_{i})+c_{2}\cdot {\vec{R}}_{2}\cdot ({\vec{P}}_{i}-{\vec{X}}_{i})$;

19: ${\vec{V}}_{i}={\vec{V}}_{i}\in [LS_{l},LS_{u}]$;

20: $\delta_{i}=|{\vec{X}}_{i,d}-{\vec{P}}_{G,i}|$;

21: $U_{i}(\delta_{i})=\{\begin{array}{cc} 1, & \delta_{i}\leq \delta^{\prime }\\ 0, & \delta_{i}>\delta^{\prime } \end{array}$;

22: ${\vec{X}}_{i}=(1-U_{i})\cdot ({\vec{X}}_{i}+{\vec{V}}_{i})+U_{i}\cdot \Psi_{i}$;

23: ${\vec{X}}_{i,d}=\{\begin{array}{cc} \max(L_{l},L_{u}+(L_{u}-{\vec{X}}_{i,d})), & {\vec{X}}_{i,d}>L_{u}\\ \min(L_{u},L_{l}+(L_{u}-{\vec{X}}_{i,d})), & {\vec{X}}_{i,d}<L_{l} \end{array}$;

24: ${\vec{X}}_{i}={\vec{X}}_{i}\in [L_{l},L_{u}]$;

25: $C_{i}=f({\vec{X}}_{i})$;

26: **for** i=1 to n **do**

27:  **if** $C_{i}<G_{P,i}$ **then**

28:   $G_{P,i}=C_{i}$;

29:   ${\vec{P}}_{i}={\vec{X}}_{i}$;

30:  **end** **if**

31:  **if** $G_{P,i}<G_{B}$ **then**

32:   $G_{B}=G_{P,i}$;

33:   ${\vec{P}}_{G}={\vec{P}}_{i}$;

34:  **end** **if**

35: **end** **for**

36: it=it+1;

37: **if** $\left|G_{B}(k)-G_{B}(k-1)|\leq \zeta \cdot G_{B}(k\right)$ **then**

38:  **if** $\delta <\delta_{\max}$ **then**

39:   $\delta =\delta +\delta_{\max}\cdot S_{s}$;

40:  **else**

41:   $\delta =\delta_{\min}$;

42:   **if** $S_{s}\geq S_{\max}$ **then**

43:    $S_{s}=S_{\min}$;

44:   **else**

45:    $S_{s}=S_{s}+S_{\min}$;

46:   **end** **if**

47:  **end** **if**

48: **else**

49: $\delta =\delta_{\min}$;

50: $S_{s}=S_{\min}$;

51: **end** **if**

52: $\delta_{\max}=\max({\vec{P}}_{G})\cdot fd_{\max}$;

53: $\delta_{\min}=\min({\vec{P}}_{G})\cdot fd_{\min}$;

54: $LS_{u}=\frac{\delta }{\delta_{\max}}+\frac{S_{s}}{S_{\max}}$;

55: $LS_{l}=-LS_{u}$;

56: **end while**

The original cost function in Equation 8 used in DSRegPSOP for symbolic regression includes mean square error with two penalty terms, $p_{1}$ and $p_{2}$, the first one with gain $\gamma_{1}$ penalizes the difference between the maximum and the minimum values of the predicted output $\hat{y}$ to avoid constant solutions, while the second one with gain $\gamma_{2}$ penalizes the number of times that a previous level equation is not in the current level. This forces the model to use previous levels in each level.

$$f\left({\vec{M}}_{i}\right)=\frac{1}{N}\sum_{n=1}^{N}{\left(y-\hat{y}\right)}^{2}+p_{1}+p_{2}$$ (8)

Where $p_{1}$ and $p_{2}$ are calculated with Equations 9 and 10, respectively.

$$p_{1}=\frac{\gamma_{1}}{\left|\min(\hat{y})-\max(\hat{y})\right|}$$ (9)

$$p_{2}=\gamma_{2}\cdot \left|(n_{l}-1)-\sum_{l_{0}=0}^{n_{l}-1}\Phi_{lv}\right|$$ (10)

Where $\Phi_{lv}$ is one if a previous levels is in the final level and zero if not.

The predicted output $\hat{y}$ depands from all the levels in M and is obtained by thresholding the last mathematical expression mapped to ranges [0,1], with a threshold of 0.5 for probability classification problems. The sigmoid function in Equation 11 is used to map the output to the desired ranges.

$$\hat{y}=\frac{1}{1+e^{-M_{i=n_{l}}}}$$ (11)

### 2.3 Balancing techniques for imbalanced datasets

Datasets are imbalanced because the classes are not equally represented, leading to biased models that favor the majority class. There are several strategies to deal with this issue effectively (25).

The process of balancing the dataset must use only the training set after splitting the dataset into training, validation and testing sets to avoid data leakage, which is the unintentional sharing of information between the training and testing datasets (26). Leakage inflates performance metrics making a model unreliable under real conditions (27). Moreover, since the models’ hyperparameters are optimized using k-fold cross-validation and the validation dataset is not resampled, the models are evaluated on imbalanced data, leading them to learn to ignore the minority class. Therefore, F1-score should be used as part of the cost function in the hyperparameter optimization, since it considers both precision and recall, which are essential metrics for imbalanced datasets (28, 29). Therefore, the cost function used in this work implements F1-score together with MSE and the original penalties in DSRegPSOP as defined in Equation 12. We decided maintaining MSE with reduced importance controlled with $\gamma_{3}$ in the cost funtion instead of replacing it with F1-score since it is more sensitive to small changes in the predicted values, because its determined before thresholding predictions.

$$f\left({\vec{M}}_{i}\right)=(1-\text{F1-score})+\gamma_{3}\times \text{MSE}+p_{1}+p_{2}$$ (12)

This research explores four balancing techniques using a cost function based on F1-score on the Tlalpan 2020 dataset: Oversample, Smote, Borderline Smote, and Smote with Edited Nearest Neighbors (ENN).

#### 2.3.1 Oversample and undersample

The most used techniques for balancing datasets are oversampling and undersampling with the raw dataset (30). Oversampling involves randomly selecting and duplicating samples from the minority class to balance the dataset, while undersampling involves randomly removing samples from the majority class. However, making naive copies of minority-class samples can lead to overfitting, while removing majority-class samples can result in the loss of valuable information. Therefore, more sophisticated techniques that generate synthetic samples are preferred (25, 30).

#### 2.3.2 Synthetic minority over-sampling technique

The Synthetic Minority Over-sampling Technique (SMOTE) is a popular technique for balancing imbalanced datasets by generating synthetic samples for the minority class (30). SMOTE works by dividing the dataset into nearest neighbors, then selecting a random neighbor and generating a synthetic sample by interpolating between the two samples. The synthetic samples use a desired SMOTE percentage to balance the dataset. SMOTE has been shown to improve the performance of machine learning models on imbalanced datasets (30). The SMOTE algorithm was proposed and detailed in Chawla et al. (31).

#### 2.3.3 Borderline SMOTE

Borderline-SMOTE is an extension of the SMOTE algorithm that focuses on generating synthetic samples for the minority class that are close to the decision boundary between the classes (25). The algorithm works by identifying minority-class samples near the decision boundary and generating synthetic samples by interpolating between them and their nearest neighbors. This approach improves the performance of machine learning models on imbalanced datasets by focusing on the most informative samples (32).

#### 2.3.4 SMOTE with edited nearest neighbors

SMOTE with Edited Nearest Neighbors (SMOTEENN) is a technique that combines the SMOTE algorithm with the Edited Nearest Neighbors (ENN) algorithm to improve the quality of synthetic samples generated for the minority class (25). The ENN algorithm works by removing samples from the dataset whose nearest neighbors misclassify them. By combining SMOTE with ENN, the algorithm generates synthetic samples for the minority class while also removing noisy or misclassified samples from the dataset.

### 2.4 Evaluation metrics

The problem stated in this work is a binary classification task, where the goal is to predict whether a patient will develop hypertension (positive class) or not (negative class). To evaluate the performance of the predictive models, we used several metrics commonly used in binary classification tasks, including accuracy, precision, recall, specificity, F1-score, and area under the receiver operating characteristic curve (AUC-ROC) (33). These metrics are calculated based on the number of true positives (TP), true negatives (TN), false positives (FP), false negatives (FN), true positive rate (TPR), and false positive rate (FPR) produced by the model. The formulas for each metric are provided in Equations 13–18.

$$\text{Accuracy}=\frac{TP+TN}{TP+TN+FP+FN}$$ (13)

$$\text{Precision}=\frac{TP}{TP+FP}$$ (14)

$$\text{Recall}=\frac{TP}{TP+FN}$$ (15)

$$\text{Specificity}=\frac{TN}{TN+FP}$$ (16)

$$\text{F1-score}=2\cdot \frac{\text{Precision}\cdot \text{Recall}}{\text{Precision}+\text{Recall}}$$ (17)

$$\text{AUC-ROC}=\int_{0}^{1}TPR(FPR)dFPR$$ (18)

Confidence intervals for AUC-ROC were computed empirically from the distribution of results across the top 10 configurations obtained with varying random seeds, using the 95% confidence interval defined as mean±1.96×STD.

These metrics provide a comprehensive evaluation of the performance of the model, allowing us to assess its ability to correctly classify patients with and without hypertension.

## 3 Results

We performed a data split with 80% for training and 20% for testing, then a grid search was applied in training data with 10-fold cross-validation to optimize the hyperparameters of DSRegPSOP, detailed in Table 1; the other hyperparameters in Table 2 were maintained as recommended in Montes Rivera et al. (14). Regarding $\gamma_{3}$, it was fixed at 0.5 to assign middle importance to the expected MSE — which ranges between 0 and 1 after the sigmoid transformation — and full importance to the F1-score, also in the range [0,1]. This configuration allows the algorithm to make small adjustments in MSE, which is more sensitive to changes when there are no substantial changes in F1-score, effectively providing a fine-grained optimization signal during the symbolic equation search.

**Table 1:** DSRegPSOP hyperparameters and their values used in the grid search.

Hyperparameter	Symbol	Values
Number of particles	n	5, 10, 15
Number of levels	$n_{l}$	1, 2, 3, 4
Number of operators	$n_{o}$	1, 2, 3, 4
Number of groups	$n_{g}$	1, 2, 3, 4
Min limit of speed refinement	$fd_{\min}$	$1\times 10^{-3}$, $1\times 10^{-5}$, $1\times 10^{-10}$
Max speed expansion factor	$S_{\max}$	$4\times 10^{-1}$, $4\times 10^{-2}$, $4\times 10^{-3}$
Min speed expansion factor	$S_{\min}$	$1\times 10^{-1}$, $1\times 10^{-2}$, $1\times 10^{-3}$

**Table 2:** DSRegPSOP hyperparameters with fixed values.

Hyperparameter	Symbol	Value
Max function evaluations	$fv_{\max}$	$5\times 10^{4}$
Cognitive coefficient	$c_{1}$	2.0
Social coefficient	$c_{2}$	2.0
Max inertial momentum	$M_{\max}$	1.1
Speed refinement factor	λ	1.0
Convergence tolerance	ζ	$1\times 10^{-3}$
Penalty 1 gain	$\gamma_{1}$	$1\times 10^{-10}$
Penalty 2 gain	$\gamma_{2}$	$1\times 10^{4}$
Penalty 3 gain	$\gamma_{3}$	0.5
Operators	$L_{o}$	{÷,+,−,×,∧,sin(),cos(),exp()}
Variables	$L_{v}$	{X, b, e, π }

The operators selected include sine and cosine functions since, according to Fourier and Poisson series, they can represent any function with periodic finite approximations, and overshoot in discontinuities (34). Operators also include the exponential function, since solving a differential equation often involves exponential terms, as its integral is itself (35). For the variables, we used all the features in the dataset X, the constants matrix b, which is a ones matrix with data size to multiply constants, e (the base of natural logarithms), and π.

Each configuration was evaluated using undersample, oversample, SMOTE, Borderline SMOTE, and SMOTEENN balancing techniques. The best configuration was selected based on the highest average F1-score across the folds. The number of configurations evaluated per balancing technique was 1,728 with a total of 8,640 configurations.

The features used were limited to those with a Pearson correlation coefficient greater than 0.05 with the target variable, resulting in a total of 39 features of the 121 in the Tlalpan 2020 Cohort dataset. Then the feature patient record, with a correlation of 0.1630, was removed to avoid dependence on the patient, leaving only 38 features.

This dimensionality reduction step was necessary to make the hyperparameter optimization of DSRegPSOP feasible, as symbolic regression algorithms based on collective intelligence are computationally intensive when operating over high-dimensional feature spaces, rendering a reliable 10-fold cross-validation grid search infeasible with 121 variables.

Pearson correlation was applied as a conservative filter to remove uninformative features, not as a data-driven search for the most predictive subset (36). It is acknowledged as a limitation that this filtering was performed on the entire dataset prior to splitting; however, given the conservative threshold applied and the statistical stability of first-order correlation measures across dataset partitions of this size, the practical impact on model evaluation is considered negligible. Furthermore, DSRegPSOP inherently performs its own feature selection during the equation-building process, as evidenced by the final models using 5-25 features. The list of maintained features based on Pearson correlation coefficients is presented in Table 3.

**Table 3:** Features from Tlalpan 2020 Cohort dataset used in the hypertension prediction model.

Variable	Feature name	Code	Pearson correlation
$X_{0}$	Basal Diastolic Blood Pressure	DBP	0.4705
$X_{1}$	Basal Systolic Blood Pressure	SBP	0.4458
$X_{2}$	Body Mass Index	BMI	0.1863
$X_{3}$	Weight	Weight	0.1782
$X_{4}$	Lack of Air	Lackair	0.1639
$X_{5}$	Waist Size	Waistsize	0.1594
$X_{6}$	HDL Cholesterol	HDLcholesterol	0.1327
$X_{7}$	Leukocytes Number	LeukocytesN	0.1324
$X_{8}$	Sitting Minutes per Day (Low)	SittingminsdayLow	0.1298
$X_{9}$	Neutrophils Number	NeutrophilsN	0.1291
$X_{10}$	Triglycerides	Triglycerides	0.1245
$X_{11}$	Heart Rate	Heartrate	0.1234
$X_{12}$	Sitting Minutes per Day (High)	SittingminsdayHigh	0.1172
$X_{13}$	Glucose	Glucose	0.0983
$X_{14}$	Trait Anxiety (High)	TraitanxietyHigh	0.0878
$X_{15}$	Trait Anxiety	Traitanxiety	0.0863
$X_{16}$	Postgraduate Education	Postgraduate	0.0792
$X_{17}$	Sufficient Sleep	Sufficient	0.0782
$X_{18}$	Lymphocytes Number	LymphocytesN	0.0774
$X_{19}$	Sleep Adequacy	Sleepadequacy	0.0768
$X_{20}$	Trait Anxiety (Low)	TraitanxietyLow	0.0762
$X_{21}$	Hours to Fall Asleep	Hoursfallasleep	0.0740
$X_{22}$	Urine Creatinine	Urinecreatinine	0.0724
$X_{23}$	Monocytes Number	MonocytesN	0.0711
$X_{24}$	Passive Smoker	Passivesmoker	0.0674
$X_{25}$	Father with Hypertension	Fatherhypertension	0.0665
$X_{26}$	Sleep Needed	Sleepneeded	0.0613
$X_{27}$	State Anxiety (Low)	StateanxietyLow	0.0612
$X_{28}$	Atherogenic Index	Atherogenicindex	0.0612
$X_{29}$	Drowsiness	Drowsiness	0.0604
$X_{30}$	Elementary Education	Elementary	0.0603
$X_{31}$	Erythrocytes	Erythrocytes	0.0596
$X_{32}$	Seric Iron	Sericiron	0.0584
$X_{33}$	Mother Smoking	Mothersmoking	0.0558
$X_{34}$	Mother Diabetic	Motherdiabetic	0.0554
$X_{35}$	Hundred Cigarettes	Hundredcigarettes	0.0519
$X_{36}$	Apnea	Apnea	0.0515
$X_{37}$	Uric Acid	Uricacid	0.0506

### 3.1 Undersample results

**Figure 2:**
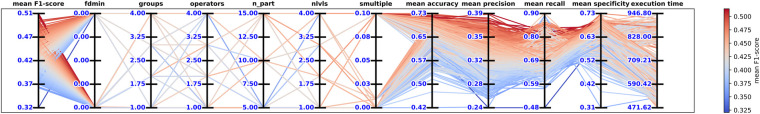
Parallelplot of all the experiments conducted using undersample balancing technique. The best configuration obtained an mean F1-score of 0.5143.

The parallelplot showing all the conducted experiments for hyperparametrization using undersample balancing technique is shown in Figure 2. The best configuration obtained a mean F1-score of 0.5143, with n=5, $n_{l}=1$, $n_{o}=4$, $n_{g}=4$, $fd_{\min}=1\times 10^{-5}$, $S_{\max}=4\times 10^{-1}$, and $S_{\min}=1\times 10^{-1}$. Table 4 shows the results of the 10 best configurations based on mean F1-score.

**Table 4:** Top 10 configurations based on mean F1-score with 10-fold cross-validation in undersample balancing.

Rank	1	2	3	4	5	6	7	8	9	10
n	5	15	10	15	15	10	10	10	5	10
$n_{g}$	4	3	1	4	3	4	3	1	2	3
$n_{o}$	4	2	4	3	2	3	1	4	4	2
$n_{l}$	1	1	1	1	3	1	4	1	1	1
$fd_{\min}$	$1\times 10^{-5}$	$1\times 10^{-5}$	$1\times 10^{-10}$	$1\times 10^{-5}$	$1\times 10^{-5}$	$1\times 10^{-10}$	$1\times 10^{-5}$	$1\times 10^{-3}$	$1\times 10^{-5}$	$1\times 10^{-3}$
$S_{\text{\textbackslash{},factor}}$	$1\times 10^{-1}$	$1\times 10^{-3}$	$1\times 10^{-1}$	$1\times 10^{-1}$	$1\times 10^{-1}$	$1\times 10^{-1}$	$1\times 10^{-1}$	$1\times 10^{-1}$	$1\times 10^{-1}$	$1\times 10^{-2}$
Mean F1-score	0.5143	0.5115	0.5104	0.5077	0.5077	0.5001	0.5001	0.4999	0.4973	0.4971
Mean accuracy	0.7105	0.7158	0.7088	0.7193	0.7228	0.6982	0.7140	0.7088	0.6947	0.7105
Mean precision	0.3904	0.3799	0.3751	0.3797	0.3834	0.3687	0.3892	0.3750	0.3654	0.3775
Mean recall	0.8185	0.8144	0.8369	0.8008	0.7867	0.8274	0.7603	0.7885	0.8268	0.7580
Mean specificity	0.6864	0.6868	0.6732	0.6959	0.7054	0.6618	0.7037	0.6874	0.6609	0.6872
Execution time (s)	535.8445	495.8413	771.9511	515.7275	569.8200	811.2351	577.6133	785.7751	511.8537	502.6398

After obtaining the 10 best hyperparameter configurations, we observed marginal differences among the mean F1-scores across configurations. To evaluate the robustness of the selected hyperparameters, each of the 10 best configurations was trained on the entire training dataset using random seeds [0,1,2,3,4,5,6,7,8,9,10], and the best model was retained. Furthermore, the maximum number of function evaluations was increased from $5\times 10^{4}$ during hyperparameter optimization to $5\times 10^{5}$ for final model training, allowing the algorithm more iterations to refine the symbolic equation once the optimal hyperparameter region had been identified. This two-stage strategy — coarse optimization followed by extended training — is a well-established practice in evolutionary and swarm intelligence algorithms, where computational resources are concentrated on the most promising configurations rather than uniformly distributed across the entire search space.

The top 10 results from undersample balancing ordered by balanced accuracy are shown in Table 5. The best model achieved a balanced accuracy of 79.23%, recall of 85.19%, specificity of 73.28%, F1-score of 56.79%, accuracy of 75.52%, and AUC-ROC of 81.27% (95% CI: 72.33%–83.87%), with a mean balanced accuracy of 75.75% ± 1.89% across the top 10 configurations. The consistency of hyperparameters across configurations indicates the robustness of the selected setup. Across all configurations explored with undersample balancing, the best individual metric values achieved were: accuracy of 78.32%, precision of 44.44%, recall of 88.89%, specificity of 82.76%, F1-score of 56.79%, balanced accuracy of 79.23%, and AUC-ROC of 82.73%.

**Table 5:** Top 10 configurations varying random seed based on balanced accuracy with undersample balancing.

Rank	1	2	3	4	5	6	7	8	9	10	Mean	STD	Best overall
Seed	1	2	4	2	6	9	3	4	6	6			
n	15	10	10	10	15	5	10	15	15	10			
$n_{g}$	4	4	1	1	4	4	3	3	3	1			
$n_{o}$	3	3	4	4	3	4	2	2	2	4			
$n_{l}$	1	1	1	1	1	1	1	3	1	1			
$fd_{\min}$	$1\times 10^{-5}$	$1\times 10^{-10}$	$1\times 10^{-3}$	$1\times 10^{-10}$	$1\times 10^{-5}$	$1\times 10^{-5}$	$1\times 10^{-3}$	$1\times 10^{-5}$	$1\times 10^{-5}$	$1\times 10^{-3}$			
$S_{\text{\textbackslash{},factor}}$	$1\times 10^{-1}$	$1\times 10^{-1}$	$1\times 10^{-1}$	$1\times 10^{-1}$	$1\times 10^{-1}$	$1\times 10^{-1}$	$1\times 10^{-2}$	$1\times 10^{-1}$	$1\times 10^{-3}$	$1\times 10^{-1}$			
Accuracy	0.7552	0.7343	0.7483	0.7063	0.7413	0.7762	0.7063	0.6713	0.6923	0.6643	0.7196	0.0372	0.7832
Precision	0.4259	0.4035	0.4151	0.3770	0.4038	0.4419	0.3729	0.3485	0.3607	0.3433	0.3893	0.0337	0.4444
Recall	0.8519	0.8519	0.8148	0.8519	0.7778	0.7037	0.8148	0.8519	0.8148	0.8519	0.8185	0.0477	0.8889
Specificity	0.7328	0.7069	0.7328	0.6724	0.7328	0.7931	0.6810	0.6293	0.6638	0.6207	0.6966	0.0533	0.8276
f1score	0.5679	0.5476	0.5500	0.5227	0.5316	0.5429	0.5116	0.4946	0.5000	0.4894	0.5258	0.0265	0.5679
Balanced accuracy	0.7923	0.7794	0.7738	0.7621	0.7553	0.7484	0.7479	0.7406	0.7393	0.7363	0.7575	0.0189	0.7923
AUC-ROC	0.8127	0.7580	0.8095	0.7725	0.8273	0.7443	0.7478	0.7711	0.7648	0.8020	0.7810	0.0295	0.8273
AUC-ROC 95% CI											[0.7233, 0.8387]		

**Figure 3:**
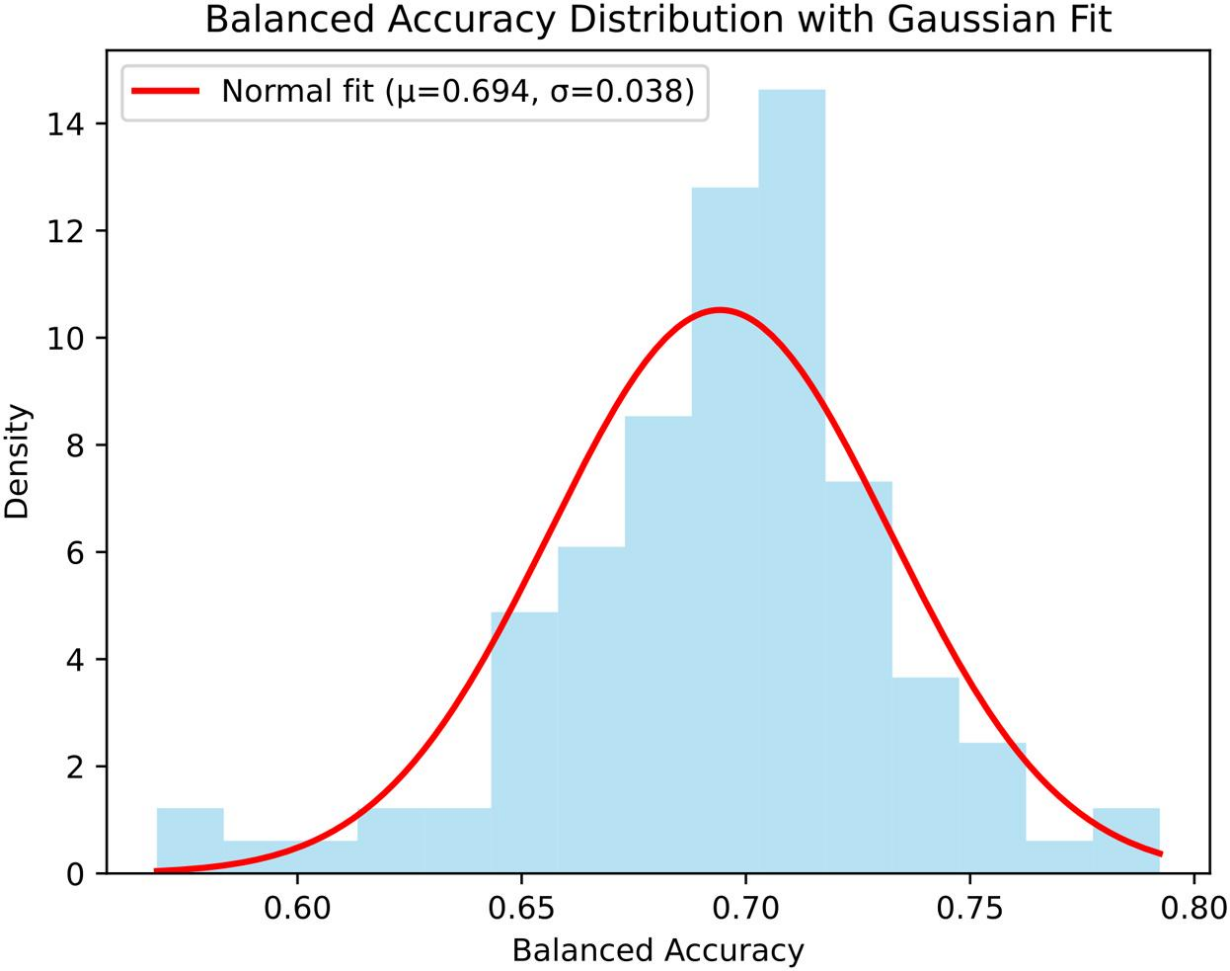
Balanced accuracy distribution of the best model with undersample balancing using different random seeds.

The Gaussian approximation of balanced accuracy distribution for the best models varying random seeds is shown in Figure 3, where the balanced accuracy mean is 0.694 and the standard deviation of 0.038, indicating that the model is robust to changes in the random seed.

The best model for hypertension prediction based on test balanced accuracy is $\text{sigmoid}(m_{0})$, if $\text{sigmoid}(m_{0})>0.5$, then hypertension is present with $m_{0}$ as in Equation 19. Despite the model is one level, we separated it into two terms for better visualization.

$$\begin{array}{rl} & m_{0}=\\ & \frac{1}{2}(X_{6}X_{19}+e^{X_{11}+X_{3}}-X_{15}X_{17}-X_{31}e^{X_{13}}-X_{19}X_{21}X_{33}+X_{20}+e^{X_{0}(X_{0}+X_{21}{)}^{X_{32}}}\\ & \;+\left|X_{6}X_{19}+e^{X_{11}+X_{3}}-X_{15}X_{17}-X_{31}e^{X_{13}}-X_{19}X_{21}X_{33}+X_{20}+e^{X_{0}(X_{0}+X_{21}{)}^{X_{32}}}\right|) \end{array}$$ (19)

The best features based on balanced accuracy obtained during the training of the model are Basal Diastolic Blood Pressure $X_{0}$, Weight $X_{3}$, HDL Cholesterol $X_{6}$, Heart Rate $X_{11}$, Glucose $X_{13}$, Trait Anxiety $X_{15}$, Sufficient Sleep $X_{17}$, Sleep Adequacy $X_{19}$, Trait Anxiety (Low) $X_{20}$, Hours to Fall Asleep $X_{21}$, Erythrocytes $X_{31}$, Seric Iron $X_{32}$, and Mother Smoking $X_{33}$. Therefore, the model uses 13 of the 38 features provided to predict hypertension and incorporates both physiological and lifestyle factors.

### 3.2 Oversample results

**Figure 4:**
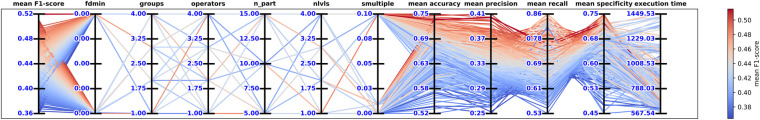
Parallelplot of all the experiments conducted using oversample balancing technique.

The parallelplot showing all the conducted experiments for hyperparametrization using oversample balancing technique is shown in Figure 4. The best configuration obtained a mean F1-score of 0.5151, with n=15, $n_{l}=3$, $n_{o}=4$, $n_{g}=4$, $fd_{\min}=1\times 10^{-3}$, $S_{\max}=4\times 10^{-1}$, and $S_{\min}=1\times 10^{-1}$. Table 6 shows the results of the 10 best configurations based on mean F1-score.

**Table 6:** Top 10 configurations based on mean F1-score with 10-fold cross-validation in oversample balancing.

Rank	1	2	3	4	5	6	7	8	9	10
n	15	15	5	15	5	5	15	10	10	10
$n_{g}$	4	4	4	4	2	3	4	4	3	4
$n_{o}$	4	2	2	1	2	2	2	1	4	2
$n_{l}$	3	3	1	2	1	1	1	1	2	2
$fd_{\min}$	$1\times 10^{-3}$	$1\times 10^{-3}$	$1\times 10^{-5}$	$1\times 10^{-5}$	$1\times 10^{-10}$	$1\times 10^{-10}$	$1\times 10^{-10}$	$1\times 10^{-10}$	$1\times 10^{-5}$	$1\times 10^{-10}$
$S_{\text{\textbackslash{},factor}}$	$1\times 10^{-1}$	$1\times 10^{-2}$	$1\times 10^{-1}$	$1\times 10^{-1}$	$1\times 10^{-1}$	$1\times 10^{-1}$	$1\times 10^{-1}$	$1\times 10^{-1}$	$1\times 10^{-3}$	$1\times 10^{-2}$
Mean F1-score	0.5151	0.5146	0.5064	0.5038	0.5025	0.5024	0.5022	0.5012	0.4990	0.4988
Mean accuracy	0.7351	0.7368	0.7456	0.7386	0.7035	0.7333	0.7491	0.7333	0.7105	0.7211
Mean precision	0.4018	0.4005	0.4065	0.3953	0.3668	0.3814	0.4014	0.3907	0.3779	0.3829
Mean recall	0.7516	0.7595	0.7124	0.7318	0.8263	0.7534	0.7138	0.7185	0.7885	0.7572
Mean specificity	0.7249	0.7306	0.7436	0.7276	0.6706	0.7205	0.7506	0.7272	0.6934	0.7116
Execution time (s)	817.10	728.10	620.13	639.04	593.38	607.31	618.47	614.22	708.80	682.08

After obtaining the 10 best hyperparameter configurations we trained each of the best 10 configurations with the entire training dataset and [0,1,2,3,4,5,6,7,8,9,10] random seeds again to evaluate robustness of the selected hyperparameters and maintained the best model. Once again, the maximum number of function evaluations was increased from $5\times 10^{4}$ during hyperparameter optimization to $5\times 10^{5}$ for final model training, allowing the algorithm more iterations to refine the symbolic equation once the optimal hyperparameter region had been identified.

The top 10 results from oversample balancing ordered by balanced accuracy are shown in Table 7. The best model achieved a balanced accuracy of 77.94%, recall of 85.19%, specificity of 70.69%, F1-score of 54.76%, accuracy of 73.43%, and AUC-ROC of 77.04% (95% CI: 74.67%–80.66%), with a mean balanced accuracy of 75.30% ± 1.32% across the top 10 configurations. The consistency of hyperparameters across configurations indicates the robustness of the selected setup. Across all configurations explored with oversample balancing, the best individual metric values achieved were: accuracy of 78.32%, precision of 45.45%, recall of 88.89%, specificity of 79.31%, F1-score of 56.34%, balanced accuracy of 77.94%, and AUC-ROC of 83.27%.

**Table 7:** Top 10 configurations varying random seed based on balanced accuracy with oversample balancing.

Rank	1	2	3	4	5	6	7	8	9	10	Mean	STD	Best overall
Seed	5	1	3	7	3	2	6	9	0	8			
n	15	5	15	10	10	10	15	15	5	5			
$n_{g}$	4	2	4	4	4	4	4	4	3	2			
$n_{o}$	2	2	2	1	2	1	2	2	2	2			
$n_{l}$	3	1	3	1	2	1	3	1	1	1			
$fd_{\min}$	$1\times 10^{-3}$	$1\times 10^{-10}$	$1\times 10^{-3}$	$1\times 10^{-10}$	$1\times 10^{-10}$	$1\times 10^{-10}$	$1\times 10^{-3}$	$1\times 10^{-10}$	$1\times 10^{-10}$	$1\times 10^{-10}$			
$S_{\text{\textbackslash{},factor}}$	$1\times 10^{-2}$	$1\times 10^{-1}$	$1\times 10^{-2}$	$1\times 10^{-1}$	$1\times 10^{-2}$	$1\times 10^{-1}$	$1\times 10^{-2}$	$1\times 10^{-1}$	$1\times 10^{-1}$	$1\times 10^{-1}$			
accuracy	0.7343	0.7832	0.7343	0.7622	0.7552	0.7273	0.6993	0.7203	0.7413	0.7413	0.7399	0.0233	0.7832
precision	0.4035	0.4545	0.4000	0.4255	0.4167	0.3889	0.3667	0.3818	0.4000	0.4000	0.4038	0.0244	0.4545
recall	0.8519	0.7407	0.8148	0.7407	0.7407	0.7778	0.8148	0.7778	0.7407	0.7407	0.7741	0.0408	0.8889
specificity	0.7069	0.7931	0.7155	0.7672	0.7586	0.7155	0.6724	0.7069	0.7414	0.7414	0.7319	0.0353	0.7931
f1score	0.5476	0.5634	0.5366	0.5405	0.5333	0.5185	0.5057	0.5122	0.5195	0.5195	0.5297	0.0178	0.5634
balanced accuracy	0.7794	0.7669	0.7652	0.7540	0.7497	0.7466	0.7436	0.7424	0.7411	0.7411	0.7530	0.0132	0.7794
AUC-ROC	0.7704	0.7851	0.8019	0.7626	0.7894	0.7609	0.7944	0.7593	0.7653	0.7771	0.7766	0.0153	0.8327
AUC-ROC 95% CI											[0.7467, 0.8066]		

**Figure 5:**
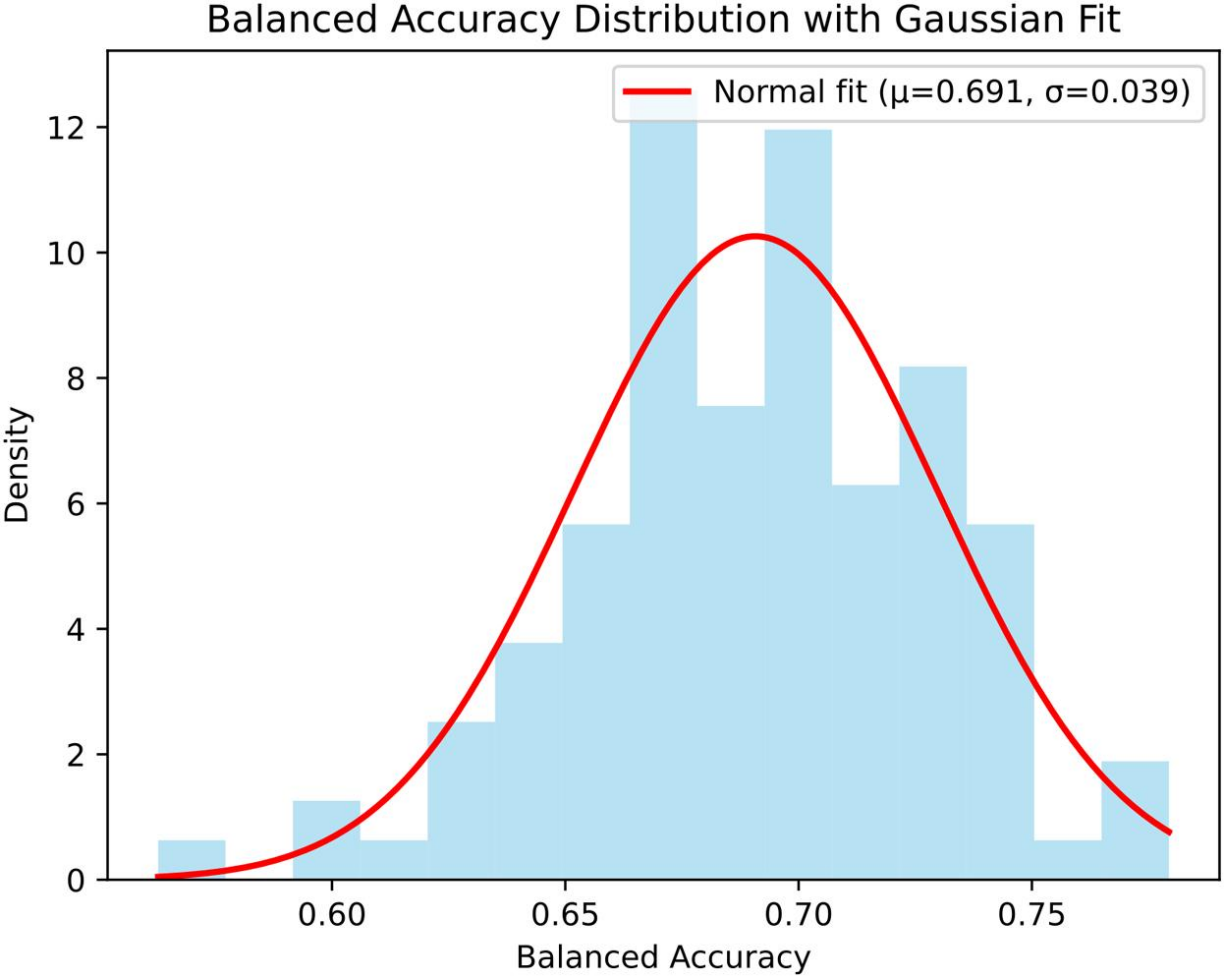
Balanced accuracy distribution of the best model with oversample balancing using different random seeds.

The Gaussian approximation of balanced accuracy distribution for the best models varying random seeds is shown in Figure 5, where the accuracy mean is 0.691 and the standard deviation of 0.039, indicating that the model is robust to changes in the random seed.

The best model for hypertension prediction based on test balanced accuracy is $\text{sigmoid}(m_{2})$, with $m_{0}$, $m_{1}$, and $m_{2}$ as in Equations 20, 21, and 22, respectively. If $\text{sigmoid}(m_{2})>0.5$, then hypertension is present. The model has three levels; each level is presented in a separate equation for better visualization.

$$\begin{array}{rl} m_{0} & =\frac{1}{2\left(X_{1}\cos(X_{29})+X_{9}\right)}[|X_{2}+X_{4}+\cos(X_{5})\\ & \;-\left(X_{1}\cos(X_{29})+X_{9}\right)\left(X_{13}X_{28}\cos(X_{7})+e^{X_{31}+\frac{\sin(X_{7})}{X_{23}}}\right)|\\ & \;-\left(X_{1}\cos(X_{29})+X_{9}\right)\left(X_{13}X_{28}\cos(X_{7})+e^{X_{31}+\frac{\sin(X_{7})}{X_{23}}}\right)\\ & \;+X_{2}+X_{4}+\cos(X_{5})] \end{array}$$ (20)

$$\begin{array}{rl} m_{1} & =\frac{1}{2\left(X_{20}+\cos^{X_{15}}(m_{0})\right)}[X_{9}^{X_{20}}\sin(X_{24})\\ & \;+|X_{9}^{X_{20}}\sin(X_{24})\\ & \;+\left(X_{20}+\cos^{X_{15}}(m_{0})\right)\left(-X_{18}X_{28}+X_{28}-e^{X_{14}X_{37}e^{X_{26}}}\right)|\\ & \;+\left(X_{20}+\cos^{X_{15}}(m_{0})\right)\left(-X_{18}X_{28}+X_{28}-e^{X_{14}X_{37}e^{X_{26}}}\right)] \end{array}$$ (21)

$$\begin{array}{cc} m_{2}=\; & \frac{1}{2X_{16}\left(X_{29}-m_{1}\sin(X_{33})\right)}[|X_{16}\left(X_{25}+e^{X_{14}+X_{15}-\sin(X_{22})}\right)\left(X_{29}-m_{1}\sin(X_{33})\right)\;\\ \; & +X_{16}\left(X_{0}e^{X_{30}}+X_{21}\right)\;\\ \; & -\left(X_{29}-m_{1}\sin(X_{33})\right)\cos(X_{30})|\;\\ \; & -X_{16}\left(X_{25}+e^{X_{14}+X_{15}-\sin(X_{22})}\right)\left(X_{29}-m_{1}\sin(X_{33})\right)\;\\ \; & -X_{16}\left(X_{0}e^{X_{30}}+X_{21}\right)\;\\ \; & +\left(X_{29}-m_{1}\sin(X_{33})\right)\cos(X_{30})]\; \end{array}$$ (22)

The model for hypertension prediction is $\text{sigmoid}(m_{2})$, with $m_{2}$ as in Equation 22, $m_{1}$ as in Equation 21, and $m_{0}$ as in Equation 20. If $\text{sigmoid}(m_{2})>0.5$, then hypertension is present. The best features obtained during the training of the model are Basal Diastolic Blood Pressure $X_{0}$, Basal Systolic Blood Pressure $X_{1}$, Body Mass Index $X_{2}$, Lack of Air $X_{4}$, Waist Size $X_{5}$, Leukocytes Number $X_{7}$, Neutrophils Number $X_{9}$, Glucose $X_{13}$, Trait Anxiety (High) $X_{14}$, Trait Anxiety $X_{15}$, Postgraduate Education $X_{16}$, Lymphocytes Number $X_{18}$, Trait Anxiety (Low) $X_{20}$, Hours to Fall Asleep $X_{21}$, Urine Creatinine $X_{22}$, Monocytes Number $X_{23}$, Passive Smoker $X_{24}$, Father with Hypertension $X_{25}$, Sleep Needed $X_{26}$, Atherogenic Index $X_{28}$, Drowsiness $X_{29}$, Elementary Education $X_{30}$, Erythrocytes $X_{31}$, Mother Smoking $X_{33}$, and Uric Acid $X_{37}$. Therefore, the model uses 25 of the 38 features provided to predict hypertension and incorporates both physiological and lifestyle factors.

### 3.3 SMOTE results

**Figure 6:**
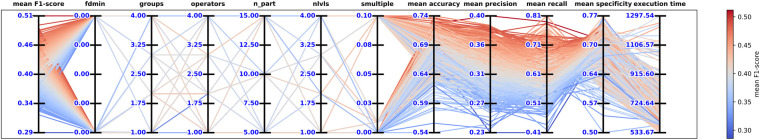
Parallelplot of all the experiments conducted using smote balancing technique.

The parallelplot showing all the conducted experiments for hyperparametrization using smote balancing technique is shown in Figure 6.

The best configuration obtained a mean F1-score of 0.5118, with n=5, $n_{l}=3$, $n_{o}=1$, $n_{g}=3$, $fd_{\min}=1\times 10^{-3}$, $S_{\max}=4\times 10^{-3}$, and $S_{\min}=1\times 10^{-3}$. Table 8 shows the results of the 10 best configurations based on mean F1-score.

**Table 8:** Top 10 configurations based on mean F1-score with 10-fold cross-validation in smote balancing.

Rank	1	2	3	4	5	6	7	8	9	10
n	5	15	15	15	10	10	5	15	15	10
$n_{g}$	3	3	2	3	3	2	4	2	3	3
$n_{o}$	1	2	2	4	1	4	2	2	2	1
$n_{l}$	3	1	3	1	2	4	2	4	1	3
$fd_{\min}$	$1\times 10^{-3}$	$1\times 10^{-3}$	$1\times 10^{-10}$	$1\times 10^{-10}$	$1\times 10^{-10}$	$1\times 10^{-3}$	$1\times 10^{-5}$	$1\times 10^{-3}$	$1\times 10^{-10}$	$1\times 10^{-10}$
$S_{\text{\textbackslash{},factor}}$	$1\times 10^{-3}$	$1\times 10^{-2}$	$1\times 10^{-3}$	$1\times 10^{-1}$	$1\times 10^{-1}$	$1\times 10^{-2}$	$1\times 10^{-2}$	$1\times 10^{-1}$	$1\times 10^{-1}$	$1\times 10^{-2}$
Mean F1-score	0.5118	0.4940	0.4891	0.4859	0.4851	0.4841	0.4836	0.4827	0.4824	0.4817
Mean accuracy	0.7263	0.7105	0.7228	0.7298	0.7263	0.7333	0.7193	0.7351	0.7333	0.7246
Mean precision	0.3998	0.3744	0.3793	0.3813	0.3779	0.3761	0.3753	0.3831	0.3848	0.3829
Mean recall	0.7853	0.7789	0.7357	0.7050	0.7073	0.7049	0.7319	0.6812	0.6784	0.6951
Mean specificity	0.7157	0.6969	0.7198	0.7292	0.7212	0.7313	0.7168	0.7398	0.7393	0.7306
Execution time (s)	654.77	611.44	636.44	623.00	614.05	735.26	661.24	663.43	615.21	654.80

After obtaining the 10 best hyperparameter configurations for smote we trained with the entire training dataset and [0,1,2,3,4,5,6,7,8,9,10] random seeds again to evaluate robustness of the selected hyperparameters and maintained the best model. Once again, the maximum number of function evaluations was increased from $5\times 10^{4}$ during hyperparameter optimization to $5\times 10^{5}$ for final model training, allowing the algorithm more iterations to refine the symbolic equation once the optimal hyperparameter region had been identified.

The top 10 results from SMOTE balancing ordered by balanced accuracy are shown in Table 9. The best model achieved a balanced accuracy of 77.81%, recall of 81.48%, specificity of 74.14%, F1-score of 55.70%, accuracy of 75.52%, and AUC-ROC of 77.12% (95% CI: 68.69%–85.54%), with a mean balanced accuracy of 74.57% ± 1.53% across the top 10 configurations. The consistency of hyperparameters across configurations indicates the robustness of the selected setup. Across all configurations explored with SMOTE balancing, the best individual metric values achieved were: accuracy of 81.82%, precision of 51.72%, recall of 85.19%, specificity of 87.93%, F1-score of 56.25%, balanced accuracy of 77.81%, and AUC-ROC of 86.91%.

**Table 9:** Top 10 configurations varying random seed based on balanced accuracy with smote balancing.

Rank	1	2	3	4	5	6	7	8	9	10	Mean	STD	Best overall
Seed	6	3	10	2	5	4	6	4	6	3			
n	15	5	15	15	5	5	15	15	5	10			
$n_{g}$	3	4	2	3	4	4	2	3	4	3			
$n_{o}$	4	2	2	2	2	2	2	2	2	1			
$n_{l}$	1	2	4	1	2	2	4	1	2	2			
$fd_{\min}$	$1\times 10^{-10}$	$1\times 10^{-5}$	$1\times 10^{-3}$	$1\times 10^{-10}$	$1\times 10^{-5}$	$1\times 10^{-5}$	$1\times 10^{-3}$	$1\times 10^{-3}$	$1\times 10^{-5}$	$1\times 10^{-10}$			
$S_{\text{\textbackslash{},factor}}$	$1\times 10^{-1}$	$1\times 10^{-2}$	$1\times 10^{-1}$	$1\times 10^{-1}$	$1\times 10^{-2}$	$1\times 10^{-2}$	$1\times 10^{-1}$	$1\times 10^{-2}$	$1\times 10^{-2}$	$1\times 10^{-1}$			
accuracy	0.7552	0.7343	0.8042	0.7692	0.7902	0.7413	0.7552	0.7762	0.7273	0.6573	0.7510	0.0409	0.8182
precision	0.4231	0.4000	0.4865	0.4318	0.4615	0.4000	0.4130	0.4390	0.3846	0.3382	0.4178	0.0414	0.5172
recall	0.8148	0.8148	0.6667	0.7037	0.6667	0.7407	0.7037	0.6667	0.7407	0.8519	0.7370	0.0686	0.8519
specificity	0.7414	0.7155	0.8362	0.7845	0.8190	0.7414	0.7672	0.8017	0.7241	0.6121	0.7543	0.0643	0.8793
f1score	0.5570	0.5366	0.5625	0.5352	0.5455	0.5195	0.5205	0.5294	0.5063	0.4842	0.5297	0.0234	0.5625
balanced accuracy	0.7781	0.7652	0.7514	0.7441	0.7428	0.7411	0.7355	0.7342	0.7324	0.7320	0.7457	0.0153	0.7781
AUC-ROC	0.7712	0.8691	0.7633	0.7356	0.7261	0.7656	0.7363	0.7527	0.7746	0.8167	0.7711	0.0430	0.8691
AUC-ROC 95% CI											[0.6869, 0.8554]		

**Figure 7:**
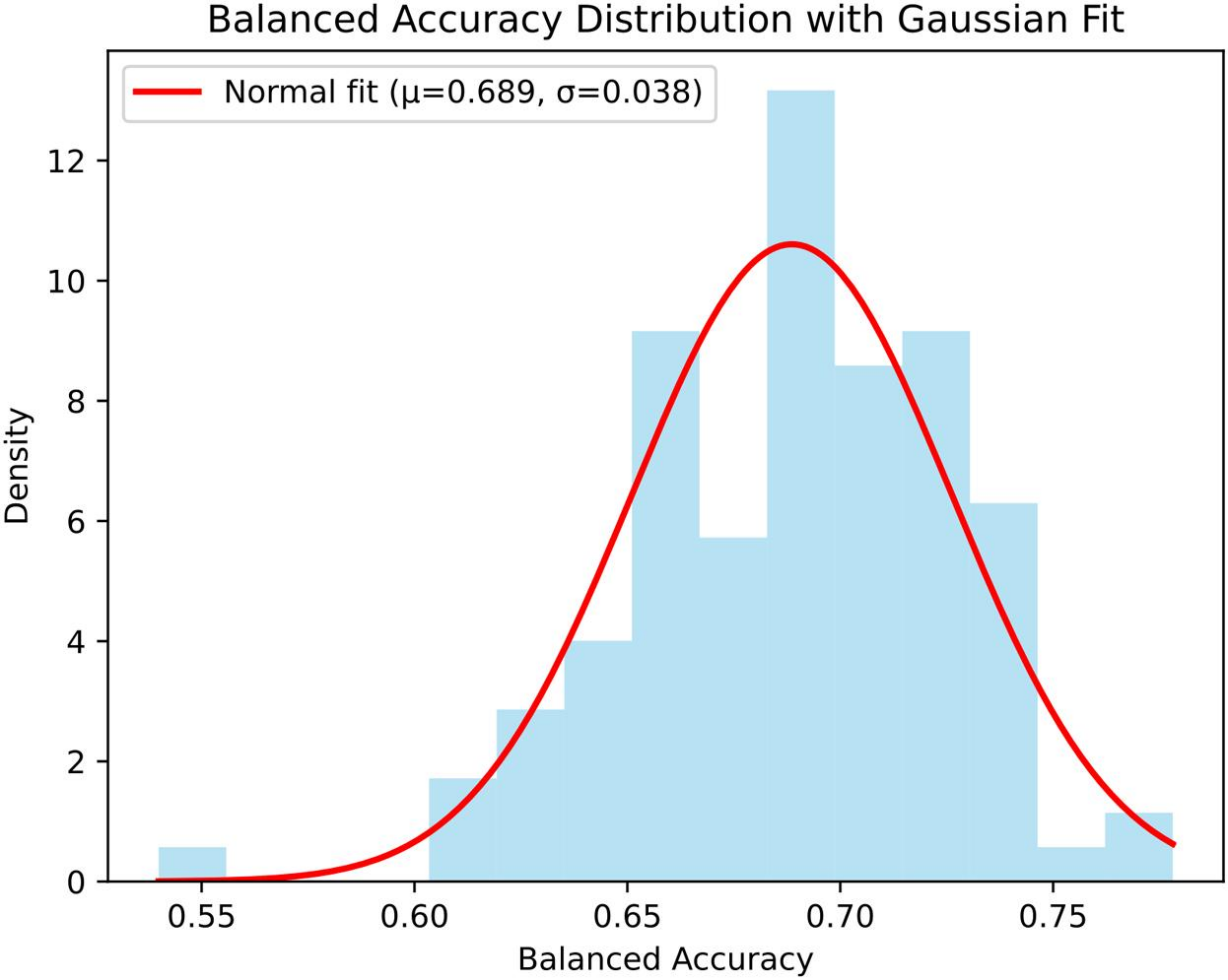
Balanced accuracy distribution of the best model with smote balancing using different random seeds.

The Gaussian approximation of balanced accuracy distribution for the best models varying random seeds is shown in Figure 7, where the accuracy mean is 0.689 and the standard deviation of 0.038, indicating that the model is robust to changes in the random seed.

The best model for hypertension prediction based on test balanced accuracy is $\text{sigmoid}(m_{0})$, where $m_{0}$ is defined in Equation 23. If $\text{sigmoid}(m_{0})>0.5$, hypertension is predicted as present. Although the model has one level, we separated it into four levels for better visualization.

$$\begin{array}{ccc} m_{0}\; & =\; & \frac{1}{2}[X_{22}X_{9}\sin(X_{26})+X_{25}+{\left(X_{0}+X_{21}\right)}^{X_{21}}.\;\\ \; & \; & +|X_{22}X_{9}\sin(X_{26})+X_{25}+{\left(X_{0}+X_{21}\right)}^{X_{21}}.\;\\ \; & \; & -e^{X_{17}}+e^{\frac{\pi }{X_{1}}}-\sin\left(X_{0}-\cos(X_{15})+e-\frac{X_{25}}{X_{11}}\right)|\;\\ \; & \; & -e^{X_{17}}+e^{\frac{\pi }{X_{1}}}-\sin\left(X_{0}-\cos(X_{15})+e-\frac{X_{25}}{X_{11}}\right)]\; \end{array}$$ (23)

The best features obtained during the training of the model are Basal Diastolic Blood Pressure $X_{0}$, Basal Systolic Blood Pressure $X_{1}$, Neutrophils Number $X_{9}$, Heart Rate $X_{11}$, Trait Anxiety $X_{15}$, Sufficient Sleep $X_{17}$, Hours to Fall Asleep $X_{21}$, Urine Creatinine $X_{22}$, Father with Hypertension $X_{25}$, and Sleep Needed $X_{26}$. Therefore, the model uses 10 of the 38 features provided to predict hypertension and incorporates both physiological and lifestyle factors.

### 3.4 Borderline SMOTE results

**Figure 8:**
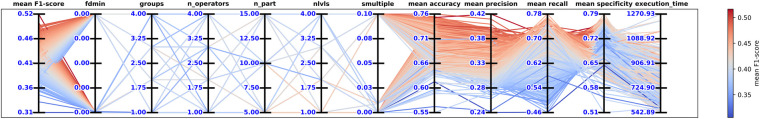
Parallelplot of all the experiments conducted using borderline smote balancing technique.

The parallelplot showing all the conducted experiments for hyperparametrization using borderline smote balancing technique is shown in Figure 8.

The best configuration obtained a mean F1-score of 0.5163, with n=10, $n_{l}=4$, $n_{o}=3$, $n_{g}=1$, $fd_{\min}=1\times 10^{-10}$, $S_{\max}=4\times 10^{-1}$, and $S_{\min}=1\times 10^{-3}$. Table 10 shows the results of the 10 best configurations based on mean F1-score.

**Table 10:** Top 10 configurations based on mean F1-score with 10-fold cross-validation in borderline-smote balancing.

Rank	1	2	3	4	5	6	7	8	9	10
n	10	5	10	10	5	5	15	10	15	10
$n_{g}$	1	4	1	4	2	4	3	3	3	1
$n_{o}$	3	1	4	1	2	4	1	1	4	2
$n_{l}$	4	2	2	2	1	1	3	3	1	2
$fd_{\min}$	$1\times 10^{-10}$	$1\times 10^{-10}$	$1\times 10^{-5}$	$1\times 10^{-3}$	$1\times 10^{-5}$	$1\times 10^{-10}$	$1\times 10^{-5}$	$1\times 10^{-5}$	$1\times 10^{-3}$	$1\times 10^{-5}$
$S_{\text{\textbackslash{},factor}}$	$1\times 10^{-3}$	$1\times 10^{-2}$	$1\times 10^{-2}$	$1\times 10^{-1}$	$1\times 10^{-2}$	$1\times 10^{-1}$	$1\times 10^{-3}$	$1\times 10^{-1}$	$1\times 10^{-3}$	$1\times 10^{-1}$
mean F1-score	0.5163	0.4982	0.4977	0.4915	0.4913	0.4900	0.4898	0.4893	0.4890	0.4890
mean accuracy	0.7491	0.7333	0.7316	0.7439	0.7561	0.7404	0.7351	0.7509	0.7246	0.7474
mean precision	0.4214	0.3960	0.3877	0.3896	0.4120	0.3828	0.3925	0.3932	0.3781	0.3764
mean recall	0.7024	0.7014	0.7386	0.6909	0.6512	0.7025	0.7062	0.6634	0.7205	0.7237
mean specificity	0.7572	0.7347	0.7305	0.7463	0.7719	0.7462	0.7432	0.7625	0.7199	0.7389
execution time (s)	649.74	654.86	614.54	640.70	649.74	640.17	654.25	653.45	593.89	716.51

After obtaining the 10 best hyperparameter configurations for borderline-smote we trained with the entire training dataset and [0,1,2,3,4,5,6,7,8,9,10] random seeds again to evaluate robustness of the selected hyperparameters and maintained the best model. Once again, the maximum number of function evaluations was increased from $5\times 10^{4}$ during hyperparameter optimization to $5\times 10^{5}$ for final model training, allowing the algorithm more iterations to refine the symbolic equation once the optimal hyperparameter region had been identified.

The top 10 results from Borderline-SMOTE balancing ordered by balanced accuracy are shown in Table 11. The best model achieved a balanced accuracy of 77.43%, recall of 70.37%, specificity of 84.48%, F1-score of 59.38%, accuracy of 81.82%, and AUC-ROC of 77.33% (95% CI: 71.45%–81.47%), with a mean balanced accuracy of 75.80% ± 0.93% across the top 10 configurations. The consistency of hyperparameters across configurations indicates the robustness of the selected setup. Across all configurations explored with Borderline-SMOTE balancing, the best individual metric values achieved were: accuracy of 81.82%, precision of 51.35%, recall of 81.48%, specificity of 86.21%, F1-score of 59.38%, balanced accuracy of 77.43%, and AUC-ROC of 81.66%.

**Table 11:** Top 10 configurations varying random seed based on balanced accuracy with borderline-smote balancing.

Rank	1	2	3	4	5	6	7	8	9	10	Mean	STD	Best overall
Seed	7	9	10	10	6	8	7	1	10	4			
n	5	10	10	5	10	10	10	15	10	10			
$n_{g}$	2	1	1	4	1	3	1	3	1	1			
$n_{o}$	2	4	2	1	4	1	3	1	3	4			
$n_{l}$	1	2	2	2	2	3	4	3	4	2			
$fd_{\min}$	$1\times 10^{-5}$	$1\times 10^{-5}$	$1\times 10^{-5}$	$1\times 10^{-10}$	$1\times 10^{-5}$	$1\times 10^{-5}$	$1\times 10^{-10}$	$1\times 10^{-5}$	$1\times 10^{-10}$	$1\times 10^{-5}$			
$S_{\text{\textbackslash{},factor}}$	$1\times 10^{-2}$	$1\times 10^{-2}$	$1\times 10^{-1}$	$1\times 10^{-2}$	$1\times 10^{-2}$	$1\times 10^{-1}$	$1\times 10^{-3}$	$1\times 10^{-3}$	$1\times 10^{-3}$	$1\times 10^{-2}$			
accuracy	0.8182	0.8042	0.8042	0.7762	0.7972	0.8112	0.7832	0.7063	0.7063	0.7273	0.7734	0.0436	0.8182
precision	0.5135	0.4872	0.4872	0.4444	0.4750	0.5000	0.4524	0.3729	0.3729	0.3889	0.4494	0.0533	0.5135
recall	0.7037	0.7037	0.7037	0.7407	0.7037	0.6667	0.7037	0.8148	0.8148	0.7778	0.7333	0.0518	0.8148
specificity	0.8448	0.8276	0.8276	0.7845	0.8190	0.8448	0.8017	0.6810	0.6810	0.7155	0.7828	0.0655	0.8621
f1score	0.5938	0.5758	0.5758	0.5556	0.5672	0.5714	0.5507	0.5116	0.5116	0.5185	0.5532	0.0296	0.5938
balanced accuracy	0.7743	0.7656	0.7656	0.7626	0.7613	0.7557	0.7527	0.7479	0.7479	0.7466	0.7580	0.0093	0.7743
AUC-ROC	0.7733	0.7621	0.7561	0.8166	0.7698	0.7561	0.7308	0.7383	0.7939	0.7490	0.7646	0.0256	0.8166
AUC-ROC 95% CI											[0.7145, 0.8147]		

**Figure 9:**
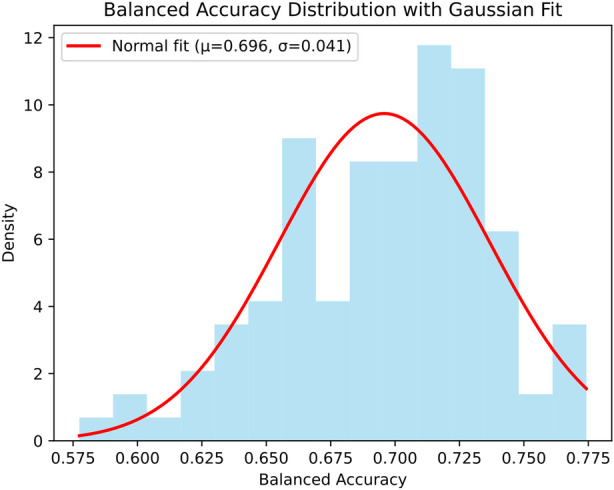
Balanced accuracy distribution of the best model with borderline-smote balancing using different random seeds.

The Gaussian approximation of balanced accuracy distribution for the best models varying random seeds is shown in Figure 9, where the accuracy mean is 0.696 and the standard deviation of 0.041, indicating that the model is robust to changes in the random seed.

The hypertension prediction model is $\text{sigmoid}(m_{0})$, where $m_{0}$ is defined in Equation 24 as the positive part of $X_{0}^{\pi }+X_{11}+X_{15}e^{X_{13}}-X_{17}$. If $\text{sigmoid}(m_{0})>0.5$, hypertension is predicted as present.

$$m_{0}=\frac{X_{0}^{\pi }+X_{11}+X_{15}e^{X_{13}}-X_{17}+\left|X_{0}^{\pi }+X_{11}+X_{15}e^{X_{13}}-X_{17}\right|}{2}$$ (24)

The features used in Equation 24 are Basal Diastolic Blood Pressure $X_{0}$, Heart Rate $X_{11}$, Glucose $X_{13}$, Trait Anxiety $X_{15}$, and Sufficient Sleep $X_{17}$. Therefore, the model uses 5 of the 38 features provided to predict hypertension and incorporates both physiological and lifestyle factors.

### 3.5 SMOTEENN results

**Figure 10:**
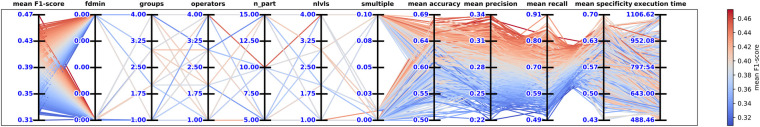
Parallelplot of all the experiments conducted using smoteenn balancing technique.

The parallelplot showing all the conducted experiments for hyperparametrization using smoteenn balancing technique is shown in Figure 10.

The best configuration obtained a mean F1-score of 0.4726, with n=5, $n_{l}=1$, $n_{o}=3$, $n_{g}=2$, $fd_{\min}=1\times 10^{-5}$, $S_{\max}=4\times 10^{-1}$, and $S_{\min}=1\times 10^{-1}$. Table 12 shows the results of the 10 best configurations based on mean F1-score.

**Table 12:** Top 10 configurations based on mean F1-score with 10-fold cross-validation in smoteenn balancing.

Rank	1	2	3	4	5	6	7	8	9	10
n	5	10	5	5	5	10	10	10	15	5
$n_{g}$	2	3	1	4	2	2	4	4	3	3
$n_{o}$	3	3	4	3	2	4	4	2	1	3
$n_{l}$	1	1	3	1	1	1	4	3	2	4
$fd_{\min}$	$1\times 10^{-5}$	$1\times 10^{-5}$	$1\times 10^{-10}$	$1\times 10^{-3}$	$1\times 10^{-3}$	$1\times 10^{-3}$	$1\times 10^{-10}$	$1\times 10^{-5}$	$1\times 10^{-3}$	$1\times 10^{-3}$
$S_{\text{\textbackslash{},factor}}$	$1\times 10^{-1}$	$1\times 10^{-2}$	$1\times 10^{-1}$	$1\times 10^{-1}$	$1\times 10^{-2}$	$1\times 10^{-1}$	$1\times 10^{-3}$	$1\times 10^{-2}$	$1\times 10^{-1}$	$1\times 10^{-1}$
mean F1-score	0.4726	0.4596	0.4575	0.4572	0.4545	0.4537	0.4522	0.4522	0.4517	0.4510
mean accuracy	0.6632	0.6649	0.6474	0.6772	0.6368	0.6579	0.6807	0.6842	0.6614	0.6579
mean precision	0.3382	0.3386	0.3301	0.3403	0.3270	0.3422	0.3434	0.3415	0.3259	0.3332
mean recall	0.8263	0.7606	0.7992	0.7355	0.8075	0.7630	0.7199	0.7077	0.7700	0.7640
mean specificity	0.6220	0.6390	0.6131	0.6552	0.5931	0.6311	0.6716	0.6783	0.6337	0.6348
execution time (s)	557.85	752.97	583.32	557.07	527.02	742.83	775.01	959.95	545.31	697.94

After obtaining the 10 best hyperparameter configurations for smoteenn we trained with the entire training dataset and [0,1,2,3,4,5,6,7,8,9,10] random seeds again to evaluate robustness of the selected hyperparameters and maintained the best model. Once again, the maximum number of function evaluations was increased from $5\times 10^{4}$ during hyperparameter optimization to $5\times 10^{5}$ for final model training, allowing the algorithm more iterations to refine the symbolic equation once the optimal hyperparameter region had been identified.

The top 10 results from SMOTEENN balancing ordered by balanced accuracy are shown in Table 13. The best model achieved a balanced accuracy of 72.08%, recall of 77.78%, specificity of 66.38%, F1-score of 48.28%, accuracy of 68.53%, and AUC-ROC of 73.18% (95% CI: 63.87%–83.07%), with a mean balanced accuracy of 69.89% ± 1.23% across the top 10 configurations. The consistency of hyperparameters across configurations indicates the robustness of the selected setup. Across all configurations explored with SMOTEENN balancing, the best individual metric values achieved were: accuracy of 68.53%, precision of 35.00%, recall of 92.59%, specificity of 66.38%, F1-score of 48.28%, balanced accuracy of 72.08%, and AUC-ROC of 82.55%.

**Table 13:** Top 10 configurations varying random seed based on balanced accuracy with SMOTEENN balancing.

Rank	1	2	3	4	5	6	7	8	9	10	Mean	STD	Best overall
Seed	4	4	8	0	5	8	6	10	9	2			
n	10	5	5	10	5	15	10	10	15	10			
$n_{g}$	4	4	2	3	2	3	4	2	3	4			
$n_{o}$	2	3	2	3	2	1	4	4	1	4			
$n_{l}$	3	1	1	1	1	2	4	1	2	4			
$fd_{\min}$	$1\times 10^{-5}$	$1\times 10^{-3}$	$1\times 10^{-3}$	$1\times 10^{-5}$	$1\times 10^{-3}$	$1\times 10^{-3}$	$1\times 10^{-10}$	$1\times 10^{-3}$	$1\times 10^{-3}$	$1\times 10^{-10}$			
$S_{\text{\textbackslash{},factor}}$	$1\times 10^{-2}$	$1\times 10^{-1}$	$1\times 10^{-2}$	$1\times 10^{-2}$	$1\times 10^{-2}$	$1\times 10^{-1}$	$1\times 10^{-3}$	$1\times 10^{-1}$	$1\times 10^{-1}$	$1\times 10^{-3}$			
accuracy	0.6853	0.6084	0.5734	0.6503	0.6434	0.6434	0.6643	0.5664	0.5594	0.6503	0.6245	0.0445	0.6853
precision	0.3500	0.3117	0.2976	0.3231	0.3182	0.3182	0.3279	0.2892	0.2857	0.3175	0.3139	0.0192	0.3500
recall	0.7778	0.8889	0.9259	0.7778	0.7778	0.7778	0.7407	0.8889	0.8889	0.7407	0.8185	0.0708	0.9259
specificity	0.6638	0.5431	0.4914	0.6207	0.6121	0.6121	0.6466	0.4914	0.4828	0.6293	0.5793	0.0700	0.6638
f1score	0.4828	0.4615	0.4505	0.4565	0.4516	0.4516	0.4545	0.4364	0.4324	0.4444	0.4522	0.0139	0.4828
balanced accuracy	0.7208	0.7160	0.7087	0.6992	0.6949	0.6949	0.6936	0.6901	0.6858	0.6850	0.6989	0.0123	0.7208
AUC-ROC	0.7318	0.7238	0.7912	0.6823	0.6804	0.7739	0.6944	0.8255	0.7011	0.6944	0.7347	0.0490	0.8255
AUC-ROC 95% CI											[0.6387, 0.8307]		

**Figure 11:**
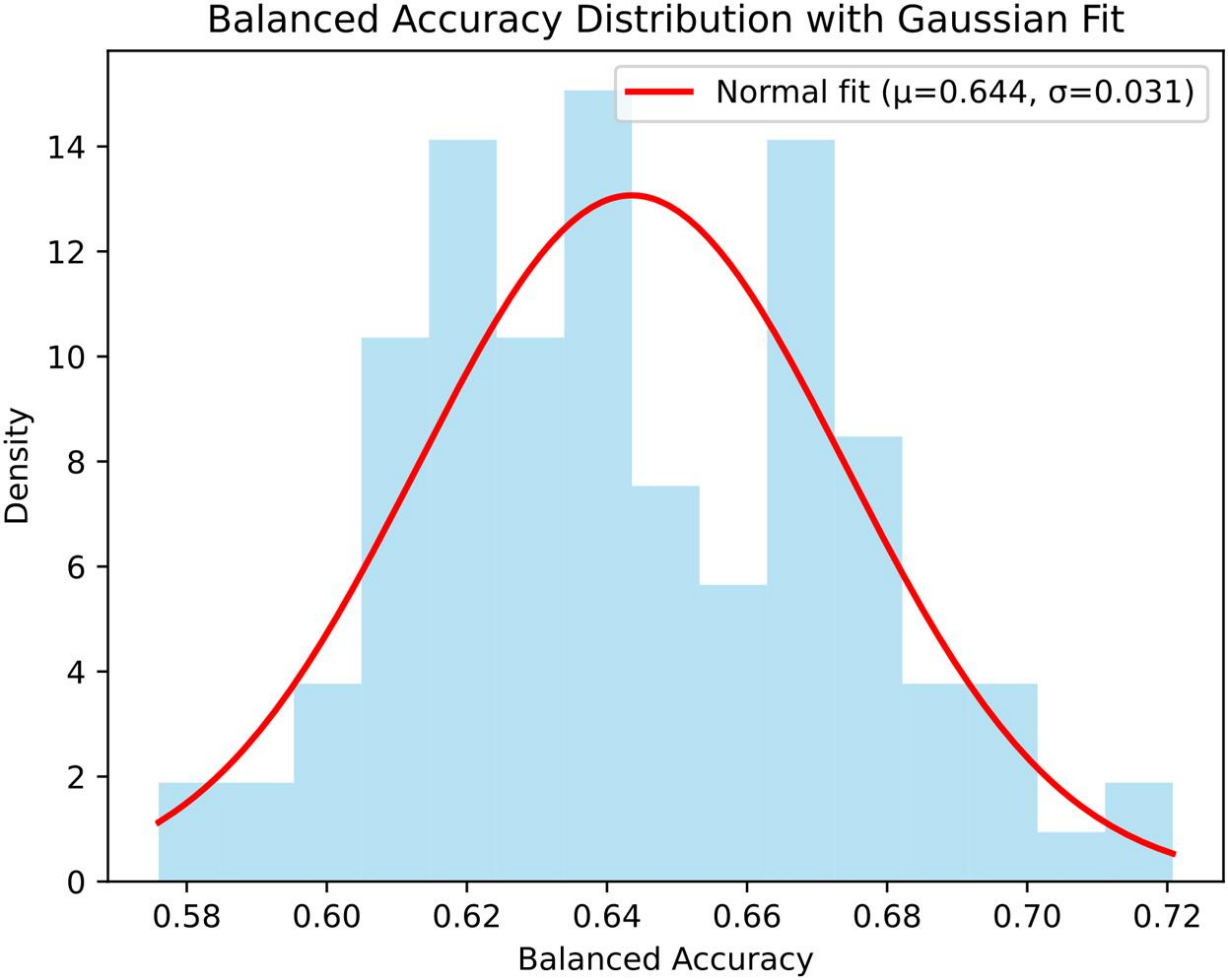
Balanced accuracy distribution of the best model with SMOTEENN balancing using different random seeds.

The Gaussian approximation of balanced accuracy distribution for the best models varying random seeds is shown in Figure 11, where the accuracy mean is 0.644 and the standard deviation of 0.031, indicating that the model is robust to changes in the random seed.

The hypertension prediction model is $\text{sigmoid}(m_{2})$, where $m_{0}$, $m_{1}$, and $m_{2}$ are intermediate functions defined in Equations 25, 26, and 27 respectively. If $\text{sigmoid}(m_{2})>0.5$, hypertension is predicted as present.

$$\begin{array}{rl} m_{0} & =X_{15}+X_{16}\sin\left(X_{18}\right)+\left(X_{0}-X_{37}\exp(X_{21})\right)\sin\left(X_{33}+\cos(X_{30}X_{5})\right)\\ & \;+\exp\left(\frac{X_{19}}{\pi X_{12}}\right) \end{array}$$ (25)

$$\begin{array}{rl} m_{1} & =X_{15}+X_{26}\sin^{X_{0}}\left(X_{14}\right)\sin\left(X_{24}\right)+X_{33}\\ & \;+e^{m_{0}-e^{X_{26}X_{4}}}+\cos\left(X_{26}\right) \end{array}$$ (26)

$$\begin{array}{rl} m_{2} & =X_{15}e^{X_{27}^{X_{29}}}+\left(X_{14}-X_{31}X_{7}\right)\sin\left(X_{12}^{X_{1}}e^{X_{33}}\right)\\ & \;+e^{m_{0}m_{1}+\sin\left(X_{31}\right)} \end{array}$$ (27)

The features used in the model are derived from Equations 25, 26, and 27 for $m_{0}$, $m_{1}$, and $m_{2}$, respectively, and include Basal Diastolic Blood Pressure $X_{0}$, Basal Systolic Blood Pressure $X_{1}$, Lack of Air $X_{4}$, Waist Size $X_{5}$, Leukocytes Number $X_{7}$, Sitting Minutes per Day (High) $X_{12}$, Trait Anxiety (High) $X_{14}$, Trait Anxiety $X_{15}$, Postgraduate Education $X_{16}$, Lymphocytes Number $X_{18}$, Sleep Adequacy $X_{19}$, Hours to Fall Asleep $X_{21}$, Passive Smoker $X_{24}$, Sleep Needed $X_{26}$, State Anxiety (Low) $X_{27}$, Drowsiness $X_{29}$, Elementary Education $X_{30}$, Mother Smoking $X_{33}$, and Uric Acid $X_{37}$. Therefore, the model uses 19 of the 38 features provided to predict hypertension and incorporates a hierarchical structure combining physiological, lifestyle, and psychological factors.

## 4 Discussion

### 4.1 Quantitative and computational modeling implications

**Figure 12:**
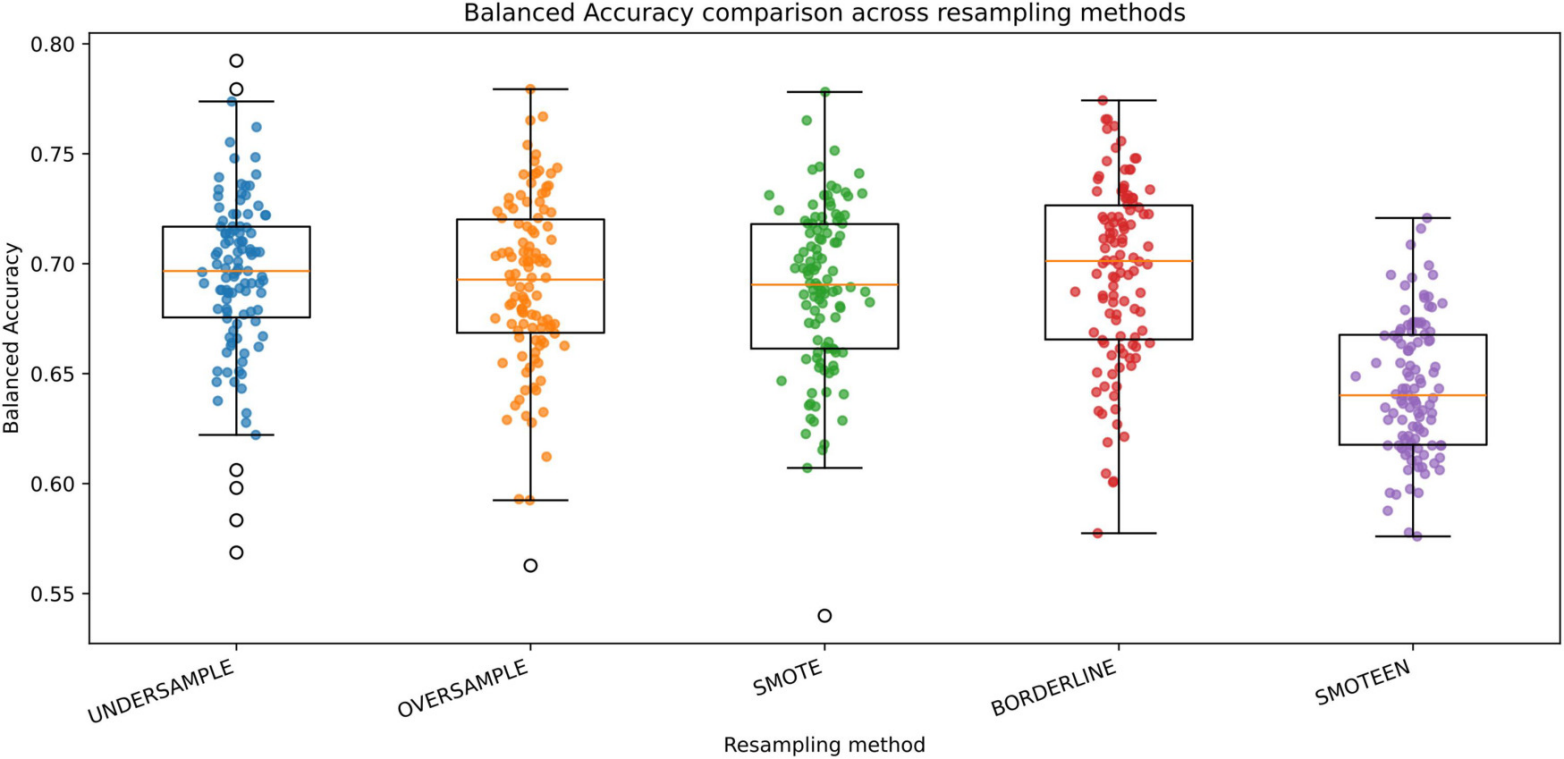
Boxplot comparing the balanced accuracy distributions using different balancing techniques.

We have developed five hypertension prediction models using different data balancing techniques: Undersample (blue), Oversample (orange), SMOTE (green), Borderline-SMOTE (red), and SMOTEENN (purple). Each model was optimized through hyperparameter tuning and evaluated for robustness across multiple random seeds (37–39). Across all configurations and balancing strategies, the best individual metric values achieved were: accuracy of 81.82% (SMOTE), precision of 51.72% (SMOTE), recall of 92.59% (SMOTEENN), specificity of 87.93% (SMOTE), F1-score of 59.38% (Borderline-SMOTE), balanced accuracy of 79.23% (Undersample), and AUC-ROC of 86.91% (SMOTE). Figure 12 shows the boxplot comparing the balanced accuracy distributions of all balancing techniques.

It is important to note that higher accuracy does not necessarily reflect better clinical performance under class imbalance. This is particularly evident when comparing metrics across balancing strategies: models that achieve high accuracy and specificity often do so at the expense of recall. For instance, the Borderline-SMOTE strategy yields the highest accuracy of 81.82% and specificity of 87.93%, but with a recall of only 70.37%, indicating a decision boundary biased toward correctly identifying non-hypertensive cases — the majority class. In contrast, the Undersample strategy, which achieves the best balanced accuracy of 79.23%, maintains a recall of 85.19% while accepting a lower specificity of 73.28%, reflecting a more appropriate trade-off for a clinical screening context where missing hypertensive cases carries greater risk than false alarms.

Although balancing techniques are applied exclusively during training to encourage the model to assign comparable importance to both classes, the test set preserves the original class distribution of the Tlalpan 2020 Cohort, which has a higher incidence of non-hypertensive samples (4:1 ratio). Consequently, despite the balancing effort, models evaluated solely on test accuracy tend to favor the majority class. This is precisely why balanced accuracy was adopted as the primary evaluation metric: it accounts for class imbalance by averaging sensitivity and specificity, penalizing models that disproportionately favor one class.

When evaluated by balanced accuracy, Borderline-SMOTE (32, 40), Undersample, Oversample, and SMOTE achieved comparable median balanced accuracies of 70.12%, 69.67%, 69.28%, and 69.05%, respectively, with no statistically significant differences among them (Holm-adjusted p>0.05, see Tables 14 and 15). Only SMOTEENN showed consistently lower performance, with a median balanced accuracy of 64.02%. The best individual model achieved a balanced accuracy of 79.23% using the Undersample strategy, with recall of 85.19%, specificity of 73.28%, F1-score of 56.79%, accuracy of 75.52%, and AUC-ROC of 81.27%.

**Table 14:** Balanced accuracy summary by resampling method.

Resampling method	n	Mean	Std	Median
Borderline-SMOTE	110	0.695778	0.040951	0.701229
Undersample	110	0.694240	0.037929	0.696679
Oversample	110	0.690739	0.038884	0.692768
SMOTE	110	0.688628	0.037613	0.690453
SMOTEENN	110	0.643545	0.030527	0.640166

**Table 15:** Post-hoc one-sided Mann–Whitney U tests (Holm-corrected) comparing Borderline-SMOTE against each other method based on balanced accuracy.

Borderline-SMOTE >	$p_{\text{one-sided}}$	$p_{\text{Holm}}$	α<0.05	Median${}_{\text{BL}}$	Median${}_{\text{other}}$
SMOTEENN	$1.24\times 10^{-18}$	$4.95\times 10^{-18}$	True	0.70	0.64
SMOTE	$6.10\times 10^{-2}$	$1.83\times 10^{-1}$	False	0.70	0.69
Oversample	$1.37\times 10^{-1}$	$2.73\times 10^{-1}$	False	0.70	0.69
Undersample	$2.80\times 10^{-1}$	$2.80\times 10^{-1}$	False	0.70	0.70

Table 14 summarizes accuracy statistics for each resampling method. The methods are sorted by mean balanced accuracy.

The Kruskal–Wallis statistic test allows determining whether there are statistically significant differences between the medians of three or more independent groups. In our case, we used it to compare the balanced accuracy distributions of the five resampling methods. The test yielded a significant result (H=124.276609, $p=6.515835\times 10^{-26}$), indicating that at least one method’s balanced accuracy distribution differs from the others. The Kruskal–Wallis statistic is computed from rank sums as $H=\frac{12}{N(N+1)}\sum_{i=1}^{k}\frac{R_{i}^{2}}{n_{i}}-3(N+1)$, where N is the total number of observations, k is the number of groups, $n_{i}$ is the sample size of group i, and $R_{i}$ is the sum of ranks for group i. Under the null hypothesis, $H∼\chi_{k-1}^{2}$ approximately, i.e., the distribution of H follows a chi-squared distribution with k−1 degrees of freedom.

Given the significant global result, we conducted post-hoc pairwise comparisons using one-sided Mann–Whitney U tests (testing whether Borderline-SMOTE > other methods). Borderline-SMOTE was selected as the reference method for this comparison as it achieved the highest mean balanced accuracy of 69.58% across all 110 configurations. Mann–Whitney U tests are non-parametric tests used to compare differences between two independent groups when the dependent variable is either ordinal or continuous, but not normally distributed. We applied Holm correction to control the family-wise error rate (41, 42). As shown in Table 15, Borderline-SMOTE significantly outperformed only SMOTEENN (Holm-adjusted $p=4.95\times 10^{-18}$), while no statistically significant differences were found between Borderline-SMOTE and SMOTE ($p=1.83\times 10^{-1}$), Oversample ($p=2.73\times 10^{-1}$), or Undersample ($p=2.80\times 10^{-1}$), suggesting that these four strategies yield comparable balanced accuracy distributions.

**Figure 13:**
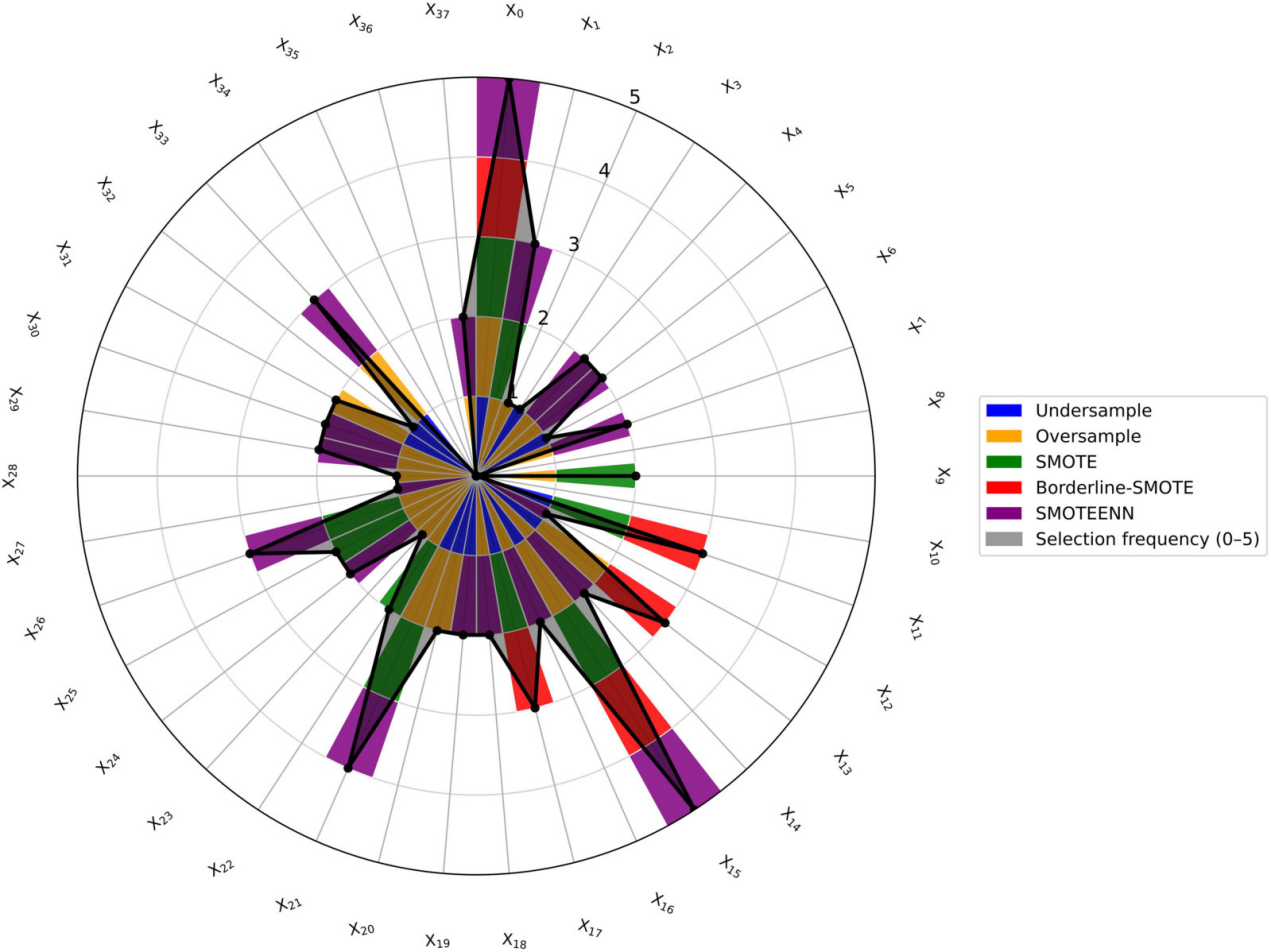
Radar plot showing the relevance of features used in different models.

To identify important features in the prediction models, we present a radar plot with features used per model in Figure 13. This plot illustrates the relevance of each feature across the different models, highlighting which features are consistently important for hypertension prediction. Notably, the 20 most relevant features across all models are Basal Diastolic Blood Pressure $X_{0}$, Trait Anxiety $X_{15}$, Hours to Fall Asleep $X_{21}$, Heart Rate $X_{11}$, Glucose $X_{13}$, Sufficient Sleep $X_{17}$, Mother Smoking $X_{33}$, Basal Systolic Blood Pressure $X_{1}$, Sleep Needed $X_{26}$, Sleep Adequacy $X_{19}$, Trait Anxiety (Low) $X_{20}$, Erythrocytes $X_{31}$, Lack of Air $X_{4}$, Waist Size $X_{5}$, Leukocytes Number $X_{7}$, Neutrophils Number $X_{9}$, Trait Anxiety (High) $X_{14}$, Postgraduate Education $X_{16}$, Lymphocytes Number $X_{18}$, and Urine Creatinine $X_{22}$.

The features found with DSRegPSOP models highlight key determinants of hypertension and, when considered jointly, support the identification of integrated clinical risk profiles to improve targeted screening and early preventive strategies (43–45).

Additionally, to validate the implicit feature selection performed by DSRegPSOP, we analyze feature importance in the Tlalpan 2020 dataset using the top-20 variables identified by three established feature selection methods — LASSO, Mutual Information, and Random Forest (Table 16). Variables marked with ⋆ correspond to those appearing in at least one of the symbolic equations generated by DSRegPSOP across the five balancing strategies. As shown, DSRegPSOP achieves 60%, 50%, and 70% coincidences with LASSO, Mutual Information, and Random Forest, respectively, demonstrating that the variables implicitly selected by symbolic regression are consistent with those identified by dedicated feature selection techniques. This result confirms that DSRegPSOP correctly identifies clinically relevant predictors of hypertension without requiring an explicit preprocessing feature selection stage, which is typically necessary for algorithms with rigid structures such as Artificial Neural Networks or Support Vector Machines.

**Table 16:** Top-20 variables ranked by LASSO, Mutual Information (MI), and Random Forest (RF). Entries marked with ⋆ indicate variables that appear in DSRegPSOP models.

Rank	LASSO	Mutual Information	Random Forest
1	$X_{0}$ ⋆	$X_{0}$ ⋆	$X_{0}$ ⋆
2	$X_{1}$ ⋆	$X_{1}$ ⋆	$X_{1}$ ⋆
3	$X_{36}$	$X_{12}$ ⋆	*MonocytesP*
4	$X_{8}$	$X_{4}$ ⋆	$X_{11}$ ⋆
5	$X_{20}$ ⋆	*Business*	$X_{2}$ ⋆
6	*Male*	$X_{5}$ ⋆	$X_{31}$ ⋆
7	$X_{33}$ ⋆	*SittingminsdayMedium*	$X_{3}$ ⋆
8	$X_{22}$ ⋆	*Fathersmoking*	$X_{9}$ ⋆
9	$X_{6}$ ⋆	*TotalmetsLow*	$X_{7}$ ⋆
10	*SittingminkweekendHigh*	$X_{9}$ ⋆	$X_{13}$ ⋆
11	$X_{28}$ ⋆	*Totalcholesterol*	$X_{6}$ ⋆
12	$X_{29}$ ⋆	$X_{2}$ ⋆	$X_{5}$ ⋆
13	*MonocytesP*	*SDIlevel*	*Hematocrit*
14	*TraitanxietyMedium*	*LymphocytesP*	$X_{32}$ ⋆
15	*Moderate*	$X_{13}$ ⋆	$X_{15}$ ⋆
16	$X_{23}$ ⋆	*Peacefulsleep*	$X_{10}$
17	$X_{12}$ ⋆	$X_{18}$ ⋆	*Totalcholesterol*
18	$X_{4}$ ⋆	*Formersmoker*	$X_{22}$ ⋆
19	*Platelets*	$X_{7}$ ⋆	*Platelets*
20	$X_{19}$ ⋆	*SDIlevel*	*Hematocrit*
Coincidence	12/20 (60%)	10/20 (50%)	14/20 (70%)

We also examined the Spearman correlation between used model features and predicted hypertension outcomes for the test dataset using the best model based on balanced accuracy, as shown in Table 17. This analysis helps to understand how each feature relates to the prediction of hypertension. Features with strong positive or negative correlations with predicted hypertension are particularly noteworthy, as they may indicate key risk factors or protective factors associated with the condition. Spearman correlation is determined with $\rho =\frac{cov(rg_{X},rg_{Y})}{\sigma_{rg_{X}}\sigma_{rg_{Y}}}$, with $rg_{X}$ and $rg_{Y}$ being the ranks of variables X and Y, respectively.

**Table 17:** Spearman correlation coefficients between model features and predicted hypertension (best model by balanced accuracy, undersample balancing).

Variable	Feature	Spearman Correlation	p-value
$X_{0}$	Basal Diastolic Blood Pressure	0.5093	$8.2523\times 10^{-11}$
$X_{3}$	Weight	0.0872	$3.0045\times 10^{-1}$
$X_{6}$	HDL Cholesterol	-0.0977	$2.4583\times 10^{-1}$
$X_{11}$	Heart Rate	0.1533	$6.7602\times 10^{-2}$
$X_{13}$	Glucose	-0.0045	$9.5702\times 10^{-1}$
$X_{15}$	Trait Anxiety	0.0450	$5.9397\times 10^{-1}$
$X_{17}$	Sufficient Sleep	0.1041	$2.1581\times 10^{-1}$
$X_{19}$	Sleep Adequacy	-0.1564	$6.2126\times 10^{-2}$
$X_{20}$	Trait Anxiety (Low)	-0.0153	$8.5605\times 10^{-1}$
$X_{21}$	Hours to Fall Asleep	0.5156	$4.4243\times 10^{-11}$
$X_{31}$	Erythrocytes	0.0973	$2.4751\times 10^{-1}$
$X_{32}$	Seric Iron	-0.0540	$5.2190\times 10^{-1}$
$X_{33}$	Mother Smoking	0.0523	$5.3498\times 10^{-1}$

It is important to note that Basal Diastolic Blood Pressure $X_{0}$ is the only blood pressure-related predictor included in this model. Moreover, the hypertension diagnosis was established at a subsequent follow-up visit and represents a longitudinal outcome rather than a concurrent measurement. We acknowledge that some participants may have presented with elevated baseline blood pressure that did not yet meet the clinical threshold at enrollment, meaning the model may partly reflect the classification of borderline cases — an inherent limitation of the cohort design. Nevertheless, the symbolic equation incorporates a broad set of predictors beyond blood pressure, including anthropometric variables ($X_{3}$), metabolic markers ($X_{6}$, $X_{13}$, $X_{31}$, $X_{32}$), inflammatory indicators ($X_{11}$), sleep-related variables ($X_{17}$, $X_{19}$, $X_{21}$), and psychosocial factors ($X_{15}$, $X_{20}$, $X_{33}$), suggesting that the model captures a comprehensive multifactorial risk profile rather than relying solely on baseline blood pressure.

### 4.2 Comparative analysis of machine learning approaches

To provide a baseline comparison, we report the performance of several machine learning models previously evaluated on the same dataset, including XGBoost, Support Vector Machines with RBF kernel, Random Forest classifiers, and logistic regression. The results are summarized in Table 18.

**Table 18:** Comparative performance of machine learning models.

Model	Balancing technique	Sensitivity	Specificity	Balanced accuracy
XGBoost	SMOTE	0.8448	0.9623	90%
XGBoost	ADASYN	0.8135	0.9677	89%
SVM - RBF	SMOTE	0.7758	0.8709	82%
SVM - RBF	ADASYN	0.8249	0.9086	87%
RF	ADASYN	0.8647	0.9846	92%
RF	SMOTE	0.8248	0.9945	90%
LR (L2)	SMOTE	0.6889	0.8167	75%
LR (L2)	ADASYN	0.7111	0.7944	75%
LR (L1)	SMOTE	0.7111	0.8278	77%
LR (L1)	ADASYN	0.7111	0.8056	76%
DSRegPSOP	Undersample	0.8519	0.7328	79%
DSRegPSOP	Oversample	0.8519	0.7069	78%
DSRegPSOP	SMOTE	0.8148	0.7414	78%
DSRegPSOP	Borderline-SMOTE	0.7037	0.8448	77%
DSRegPSOP	SMOTEENN	0.7778	0.6638	72%

Compared with logistic regression, DSRegPSOP achieves competitive or superior balanced accuracy across most balancing strategies, with the best DSRegPSOP model reaching 79% vs. 77% for the best LR (L1) configuration, and notably higher sensitivity of up to 85.19% vs. 71.11% for LR. This is particularly relevant in a clinical screening context where minimizing false negatives is a priority. It is also worth highlighting that the Tlalpan 2020 dataset presents an inherently challenging structure beyond DBP (r=0.47) and SBP (r=0.45), since all remaining features exhibit weak linear correlations with the hypertension outcome (ranging from 0.05 to 0.19), suggesting that the predictive signal is largely encoded in non-linear combinations and interactions among variables. Under these conditions, the competitive performance of DSRegPSOP relative to logistic regression is particularly noteworthy, as DSRegPSOP captures these non-linear patterns through its symbolic equation structure, while logistic regression is fundamentally constrained to linear decision boundaries. However, in datasets with stronger non-linear structure, the performance gap would be expected to widen further (14).

DSRegPSOP achieved AUC-ROC values ranging from 73% to 81% across balancing strategies, remaining within a clinically acceptable range for a hypertension screening tool. More importantly, DSRegPSOP produces explicit symbolic equations that analytically express the relationship between clinical variables and the predicted outcome, providing full mathematical transparency and direct clinical interpretability — advantages that black-box models such as XGBoost or Random Forest cannot offer even when augmented with post-hoc methods such as SHAP values. The results across all five balancing strategies also demonstrate consistent and stable behavior, as evidenced by the low standard deviations reported in Tables 5–13.

It is worth noting that while several reference models achieve higher balanced accuracy on the same dataset, DSRegPSOP offers complementary advantages that are not captured by accuracy metrics alone. The Tlalpan 2020 Cohort presents an inherently challenging predictive structure, where the signal is largely encoded in weakly correlated features (Pearson correlations ranging from 0.05 to 0.47), making it difficult for any classification method to achieve high performance across all metrics simultaneously. Under these conditions, DSRegPSOP achieves a sensitivity of up to 85.19% with a specificity of 73.28% using the Undersample strategy — a clinically relevant result for a screening tool where missing hypertensive cases carries greater risk than false alarms. Unlike black-box models such as XGBoost or Random Forest, DSRegPSOP produces explicit symbolic equations that fully express the relationship between clinical variables and the predicted outcome, providing mathematical transparency and direct clinical interpretability without requiring post-hoc explanation methods. Furthermore, DSRegPSOP performs implicit feature selection through its symbolic regression structure, eliminating the need for a dedicated preprocessing feature selection stage and reducing the risk of data leakage. In our experimental workflow, all preprocessing steps were applied exclusively within each cross-validation fold, ensuring that no information from the test set influenced model training or feature selection at any stage.

### 4.3 A clinical walkthrough of a risk model

Looking at the results of the comparative Table 18, we can see that the DSRegPSOP Undersample model achieved a good combination of sensitivity, specificity and balanced accuracy among the symbolic models. In what follows, we will present a step-by-step walkthrough example of risk calculation for a hypothetical patient. This will highlight the clinical understanding relevance of symbolic regression models.

#### 4.3.1 Hypothetical patient profile

Consider a *hypothetical patient* with the following values of the clinical parameters. All values shown are z-score normalized (mean = 0, SD = 1) as used by the model. A positive z-score indicates a value above the cohort mean; negative indicates below:

- $X_{0}$ — diastolic BP, z=+1.4 (high)
- $X_{3}$ — weight, z=+0.9
- $X_{6}$ — HDL cholesterol, z=−0.8 (low HDL)
- $X_{11}$ — heart rate, z=+0.7
- $X_{13}$ — glucose, z=+0.5
- $X_{15}$ — trait anxiety, z=+1.2 (high)
- $X_{17}$ — sufficient sleep, z=−1.0 (poor)
- $X_{19}$ — sleep adequacy, z=−0.9
- $X_{20}$ — trait anxiety (low), z=+0.3
- $X_{21}$ — hours to fall asleep, z=+1.1 (prolonged)
- $X_{31}$ — erythrocytes, z=+0.4
- $X_{32}$ — seric iron, z=+0.6
- $X_{33}$ — mother smoking, z=1 (yes)

**Figure 14:**
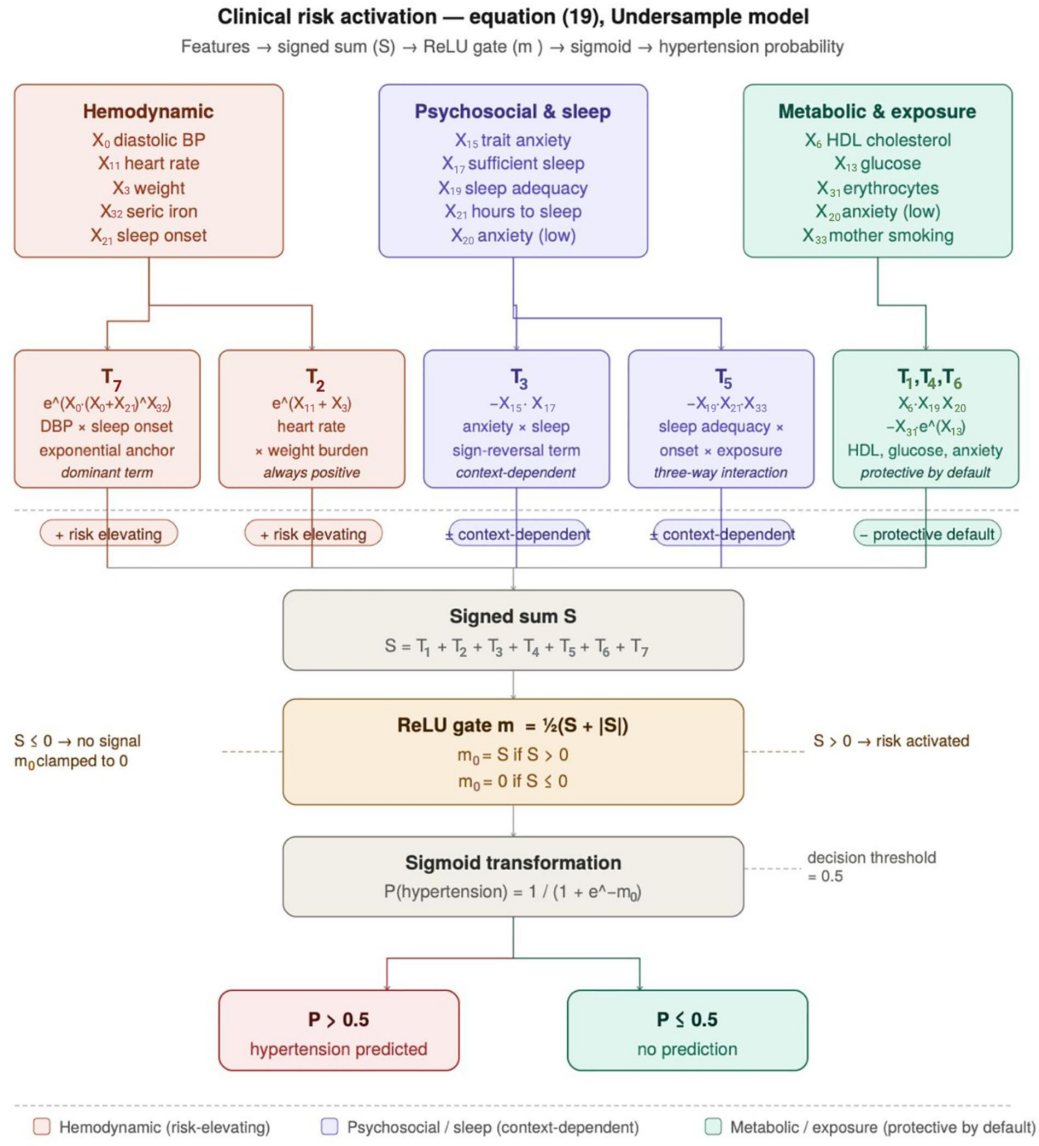
Clinical risk activation model (Figure made using Biorender.com).

We will decompose Equation 19 into a series of simpler to compute terms:

- $T_{1}=X_{6}\cdot X_{19}$
  HDL cholesterol × sleep adequacy =(−0.8)×(−0.9)=0.7200. Both are below the cohort mean (negative z-scores), making this product positive, hence **it adds to risk** here, unlike when both are favorable.
- $T_{2}=e^{\left(X_{11}+X_{3}\right)}$
  exp(heart rate + weight) = exp(0.7 + 0.9) = exp(1.60) = 4.9530. This term is always positive and **amplifies** the joint burden of elevated heart rate and higher body weight, **also adding to risk**.
- $T_{3}=-X_{15}\cdot X_{17}$
  −(trait anxiety × sufficient sleep) =−(1.2×−1)=1.2000. With high anxiety (positive) and poor sleep (negative), the product is negative, **subtracting a negative adds to risk**, as expected.
- $T_{4}=-X_{31}\cdot e^{X_{13}}$
  −(erythrocytes × exp(glucose)) =−(0.4×exp(0.5))=−(0.4×1.6487)=−0.6595. Elevated glucose amplifies this metabolic term. Because both erythrocytes and glucose are above zero, **this term is risk-reducing in direction but modest** here.
- $T_{5}=-X_{19}\cdot X_{21}\cdot X_{33}$
  −(sleep adequacy × hours to fall asleep × mother smoking) =−(−0.9×1.1×1)=0.9900. Negative sleep adequacy × positive sleep-onset latency × maternal smoking exposure (=1) yields a **positive risk contribution**.
- $T_{6}=X_{20}$ Trait anxiety (low) = 0.3000. This term enters linearly; a modestly elevated score adds a **small positive contribution**.
- $T_{7}=e^{X_{0}\cdot (X_{0}+X_{21}{)}^{X_{32}}}$
  exp[DBP × (DBP + sleep-onset)${}^{iron}$] = exp(1.4 × (1.4+1.1)${}^{0}.6$) = exp(1.4 × 1.7329) = exp(2.4260) = 11.3136. This **dominant term grows rapidly** with elevated diastolic BP and longer sleep-onset latency, reflecting their **combined hemodynamic burden**.
- Now let us sum all these contributions to calculate a risk score. Let us also recall that the model in Equation 19, used a RELU sigmoid approach to risk. $S=T_{1}+T_{2}+T_{3}+T_{4}+T_{5}+T_{6}+T_{7}=18.8172=m_{0}$
- Applying the sigmoid curve to hypertension probability:
  $P(hypertension)=\frac{1}{1+e^{-m_{0}}}=\frac{1}{1+e^{-18.8172}}≃$ 100.0%. Since this exceeds 0.5, **the model predicts hypertension as present**. Figure 14 graphically describes the entire evaluation of the model in Equation 19 for a hypothetical patient.

The terms map naturally onto distinct clinical domains as follows:

1. $T_{7}$ (the exponential DBP × sleep-onset term) is the dominant contributor, consistent with diastolic BP having the highest Pearson and Spearman correlation with outcome. Its exponential form means *the model is especially sensitive to joint elevation of DBP and sleep-onset latency, hence capturing a hemodynamic–sleep interaction*.
2. $T_{2}$ ($e^{\left(heartrate+weight\right)}$) links sympathetic burden and adiposity, both of which amplify cardiac output.
3. $T_{3}$ (-trait anxiety × sufficient sleep) is noteworthy because *when sleep is poor (negative z) and anxiety is high (positive z)*, the product is negative, thus subtracting a negative value *adds to risk*, which correctly encodes the synergy between these two risk factors.
4. $T_{5}$ (the three-way sleep adequacy × sleep-onset × maternal smoking interaction) *captures an environmental/genetic exposure modulated by sleep disturbance*.
5. $T_{1}$ (HDL × sleep adequacy) is *context-dependent: when both are favorable, it is protective; when both are impaired, it reverses direction*.

For the illustrative patient profile shown, the sigmoid output is well above 0.5 and hypertension is predicted, driven primarily by $T_{7}$ and $T_{2}$.

### 4.4 Clinical and public health implications

From a clinical perspective, the models that we have developed by means of symbolic regression highlight the multifactorial nature of early onset hypertension (46, 47). The resulting symbolic expressions can be interpreted as integrative representations of interacting physiological and behavioral processes.

The central role of hemodynamic and metabolic variables is strongly supported by the correlation analysis, where basal diastolic blood pressure and weight showed robust and statistically significant associations with predicted hypertension (ρ=0.5093 and ρ=0.0872 respectively; p<0.001) reinforces that the models are anchored in well-established clinical markers of vascular resistance and early cardiometabolic dysregulation (48–50). In addition, the models incorporate clinically meaningful non-hemodynamic factors. For example, hours to fall asleep was associated with predicted hypertension (ρ=0.5156, p<0.001), suggesting a protective role potentially mediated through autonomic balance and neuroendocrine regulation (51).

From a mechanistic perspective, the inclusion of psychosocial variables such as anxiety supports the role of chronic sympathetic activation and hypothalamic–pituitary–adrenal axis dysregulation in early blood pressure alterations (52–54). Likewise, behavioral factors such as prolonged sitting time may reflect early endothelial dysfunction and reduced metabolic efficiency (55).

One particularly striking aspect is that the models we developed allow the identification of non-linear combinations of variables (the so-called higher order effects) that may be overlooked by using traditional statistical approaches (56, 57). From the clinical standpoint, this opens the possibility of a more personalized risk evaluation (58, 59). One that considers not only isolated values but integrated patterns of exposure. The interpretability of the resulting equations facilitates their clinical validation and their potential translation to tools to support clinical decisions (2, 60, 61). This in turn, may allow the medical personnel to better understand what factors are contributing to the estimated risk and, in consequence, orient them for early specific interventions (62, 63).

To enhance clinical interpretability, we provide a simplified example based on key variables identified in the Tlalpan 2020 cohort, prioritizing those with the strongest associations with predicted hypertension. These findings can be further interpreted in terms of clinically identifiable risk profiles. Rather than reflecting isolated risk factors, the symbolic models capture combinations of hemodynamic, behavioral, and psychosocial characteristics that together define higher-risk phenotypes. This profile-based interpretation enhances the clinical applicability of the models by facilitating early identification and targeted preventive interventions.

Despite their interpretability and potential clinical utility, the implementation of these models in real world settings presents several challenges. These include the need for external validation in diverse populations, the standardization of input variables across clinical contexts, and the integration of predictive tools into existing clinical workflows (64, 65). Additionally, ensuring data quality and completeness, particularly for behavioral and psychosocial variables, remains essential for reliable deployment (66).

In comparison with established hypertension risk scores, such as those derived from Framingham based approaches, our models offer complementary advantages. Traditional risk scores typically rely on predefined linear associations between a limited set of clinical variables, primarily focused on cardiometabolic factors (67, 68). In contrast, the symbolic regression models presented here capture nonlinear relationships and incorporate a broader range of determinants, including behavioral and psychosocial factors. This may provide a more comprehensive representation of early risk, particularly in populations where traditional risk scores may have limited applicability (69).

In the context of public health, our findings have relevant implications for the design of primary prevention strategies against hypertension. Our capacity to identify individuals with higher probability to develop hypertension from routine collected information suggest that these models may be implemented in population level screening or in strategies for epidemiological surveillance (70–73). Furthermore, the use of explainable models is particularly fit for public policy contexts, in which transparency and accountability are essential to justify resource assignment and intervention prioritization (74–78).

In the context of middle-income countries such as Mexico, in which the burden of disease of chronic diseases continues increasing (79, 80), an approach such as the one presented here offers a promissory avenue to integrate advanced data science with the reals needs of health systems (81–83). By being supported in well-characterized longitudinal cohorts and interpretable methodologies, these models may contribute to reducing the gap between research and public health actions. Overall, our results support the use of explainable machine learning tools, not only as predictive instruments but also as platforms to generate actionable evidence to inform prevention policy, promotion of healthy lifestyles and the reduction of cardiovascular risks at the population level.

## 5 Conclusions

In this work we use DSRegPSOP algorithm to develop hypertension prediction models with symbolic regression approach using the Tlalpan 2020 dataset. Since the dataset is imbalanced, we applied five different data balancing techniques: Undersample, Oversample, SMOTE, Borderline-SMOTE, and SMOTEENN, with special emphasis on avoiding data leakage between training and testing sets, which is crucial for obtaining reliable model performance estimates in imbalanced datasets. Using symbolic regression allowed us to derive interpretable models that provide insights into the relationships between various features and hypertension risk and a better understanding of the underlying factors contributing to hypertension compared with black-box models.

The Undersample strategy achieved the best balanced accuracy of 79.23%, with recall of 85.19%, specificity of 73.28%, F1-score of 56.79%, and accuracy of 75.52%, followed by Oversample (77.94%), SMOTE (77.81%), Borderline-SMOTE (77.43%), and SMOTEENN (72.08%). Across all configurations and balancing strategies, the best individual metric values achieved were: accuracy of 81.82% (SMOTE), precision of 51.72% (SMOTE), recall of 92.59% (SMOTEENN), specificity of 87.93% (SMOTE), F1-score of 59.38% (Borderline-SMOTE), balanced accuracy of 79.23% (Undersample), and AUC-ROC of 86.91% (SMOTE). Figure 12 shows the boxplot comparing the balanced accuracy distributions of all balancing techniques.

We performed Kruskal–Wallis and Mann–Whitney U tests to statistically assess differences among resampling techniques based on balanced accuracy distributions. The Kruskal–Wallis test yielded H=124.276609 with $p=6.515835\times 10^{-26}$, indicating significant global differences among the methods. Subsequent Mann–Whitney U tests with Holm correction revealed that Borderline-SMOTE — selected as the reference method due to its highest mean balanced accuracy of 69.58% across all 110 configurations — significantly outperformed only SMOTEENN (Holm-adjusted $p=4.95\times 10^{-18}$), while no statistically significant differences were found between Borderline-SMOTE and SMOTE ($p=1.83\times 10^{-1}$), Oversample ($p=2.73\times 10^{-1}$), or Undersample ($p=2.80\times 10^{-1}$), suggesting that these four strategies yield comparable balanced accuracy distributions.

We also identified the contribution of features to hypertension prediction in the best model by using Spearman correlation with statistical significance supported with p-value <0.05. The features with statistically significant positive correlation to hypertension were Basal Diastolic Blood Pressure $X_{0}$ (ρ=0.5093, $p=8.25\times 10^{-11}$) and Hours to Fall Asleep $X_{21}$ (ρ=0.5156, $p=4.42\times 10^{-11}$), suggesting that elevated diastolic blood pressure and difficulty falling asleep are associated with higher likelihood of hypertension prediction.

The most relevant features across all models were Basal Diastolic Blood Pressure $X_{0}$, Trait Anxiety $X_{15}$, Hours to Fall Asleep $X_{21}$, Heart Rate $X_{11}$, Glucose $X_{13}$, Sufficient Sleep $X_{17}$, Mother Smoking $X_{33}$, Basal Systolic Blood Pressure $X_{1}$, Sleep Needed $X_{26}$, Sleep Adequacy $X_{19}$, Trait Anxiety (Low) $X_{20}$, Erythrocytes $X_{31}$, Lack of Air $X_{4}$, Waist Size $X_{5}$, Leukocytes Number $X_{7}$, Neutrophils Number $X_{9}$, Trait Anxiety (High) $X_{14}$, Postgraduate Education $X_{16}$, Lymphocytes Number $X_{18}$, and Urine Creatinine $X_{22}$. These findings highlight the importance of both physiological and lifestyle factors in hypertension risk assessment. Furthermore, the features implicitly selected by DSRegPSOP across the five balancing strategies show a 60%, 50%, and 70% coincidence with the top-20 variables identified by LASSO, Mutual Information, and Random Forest, respectively, confirming that the symbolic regression approach with DSRegPSOP consistently identifies clinically relevant predictors without requiring an explicit feature selection stage.

Despite these promising results, this study has limitations that should be acknowledged. First, the validation was performed on a single Mexican cohort (Tlalpan 2020), which may limit generalizability to other populations or geographic regions. Second, while DSRegPSOP with symbolic regression provides interpretable models, external validation in independent datasets would be essential to confirm the clinical utility of these predictions.

### 5.1 Future work

Although following rigorous procedures to avoid data leakage, hyperparameter selection, and ensure robust model evaluation, the accuracy, precision, recall, specificity, and F1-score of the models indicate room for improvement possibly working on the representativeness of the dataset. Future research could explore adding more data to the dataset with balanced data between hypertensive and non-hypertensive subjects to improve model performance. Additionally, investigating other data balancing techniques and hybrid approaches may yield further improvements in predictive accuracy. Additionally, exploring other features or combinations of features could enhance the models’ predictive capabilities. Finally, validating the models on external datasets would be essential to assess their generalizability and real-world applicability.

## Data Availability

The raw data supporting the conclusions of this article are not publicly available due to privacy and ethical restrictions, as they belong to the Tlalpan 2020 cohort. Requests to access the datasets should be directed to the corresponding author/s.
